# The dual path effect mechanism study of digital-HRM on employee innovative performance and cyberloafing

**DOI:** 10.1371/journal.pone.0307195

**Published:** 2024-08-15

**Authors:** Dongmei Hu, Yuting Lan

**Affiliations:** 1 School of Management, Xihua University, Chengdu, China; 2 Research Institute of International Economics and Management, Xihua University, Chengdu, China; University of Central Punjab, PAKISTAN

## Abstract

In recent years, an increasing number of companies have begun implementing digital-HRM. However, much of the existing research primarily discusses digital-HRM from a “thing” perspective or explores its consequences at the organizational level. There has been limited research focusing on individual employees, particularly on how digital-HRM impacts their psychological states and performance. Drawing on job demands-resources theory, this study examines the relationship between digital-HRM and employee innovative performance, as well as cyberloafing. We conducted a time-lagged study involving 487 employees across various industries in China and employed partial least squares path modeling. The results suggest that digital-HRM enhances employee innovative performance by increasing the sense of work gain, while it reduces cyberloafing by decreasing relative deprivation. Perceived ease of technology use was found to positively moderate these relationships. By rigorously investigating the critical psychological mechanisms of the sense of work gain and relative deprivation, and the essential boundary condition of perceived ease of technology use, this study aims to develop a comprehensive conceptual framework that deepens our understanding of how digital-HRM, as an emerging job resource in the digital era, influences employee behavior. Adopting a human-centered approach, the research theoretically extends the study of digital-HRM’s impact at the individual level and finds that digital-HRM influences employee performance in a mutually beneficial manner. These findings provide practical insights for organizations to actively implement digital-HRM and maximize its benefits.

## Introduction

The advent of the digital era has prompted a radical shift in human resource management (HRM), leading to a transformation from its conventional practices towards digital-HRM [1]. Scholars have extensively examined the connotation of digital-HRM and have characterized it as the socio-technical result of digitizing HRM practices [2]. Its core involves employing emerging technological methods—such as artificial intelligence, cloud computing, among others—to reimagine the employee experience through inventive approaches, and thus fosters the generation of organizational value [3, 4]. As an increasing number of enterprises embark on digital transformation, the practice of digital-HRM within organizations is set to become an irresistible trend in the coming years.

As the cornerstone of digital-HRM, the role of digital technology is undeniable. Digital technology, broadly defined as all the electronic tools, automatic systems, technological devices, and resources that generate, process, or store information in the form of binary code [5, 6], including blockchain technology, 3D printing technology, Internet of Things, 5G, cloud computing, automation and robotics, artificial intelligence and data analysis [7]. It is precisely the application of digital technology that has optimized and innovated the traditional HRM model, transforming it into digital-HRM [3, 8]. However, the impact of digital technology on organizations and individuals is multi-dimensional and complex, largely dependent on the intent and manner of its application. On one hand, digital technology can serve as a powerful assistant for employees, helping them overcome spatial and temporal constraints, enhance collaboration and cooperation efficiency, and increase their sense of self-efficacy and job satisfaction, thereby proactively improving performance [8, 9]. For instance, Rabl et al. [10] found that support from digital technology facilitates employee intrapreneurial behaviour, enabling them to exhibit behaviors beyond their roles, such as exerting extra effort. On the other hand, it may also become a foreman wielding a whip, immersing employees in pervasive digital monitoring, algorithmic control, and technological stress [11–14]. For example, Marsh et al. [15], in their review of the dark side effects of digital workplaces, noted that digital work can lead to a range of adverse consequences such as technology-related stress, overload, anxiety, interruption, distraction, addiction, and excessive use. In such environments, although employees’ performance might significantly improve, their mental health scores can substantially decline [16–18]. As found in the study by Liu et al. [19], perceived technological uncertainty can increase technology-related stress among employees, thereby threatening their subjective well-being. So, how exactly does HRM empowered by digital technology affect employee behavior and performance? Is it through technological empowerment that motivates employees to work proactively, or through digital surveillance that drives passive work? What are the psychological reactions of employees in this process? These questions remain largely unknown at present. Peccei and Van de Voorde [20] conducted a qualitative review of studies from 2000 to 2018 on the relationship between HRM, employee well-being, and organizational/individual performance, proposing three theoretical models of the HRM-WB-OIP relationship: mutual gains, conflicting outcomes, and mutual losses. They found that the mutual gains model predominates in the relationship between HRM, employee well-being, and organizational/individual performance (HRM-WB-IOP). The mutual gains model suggests that HRM should benefit both the individual and the organization [21], especially as psychological health, job satisfaction, and well-being are increasingly emphasized in the workplace [22–28], understanding which model (mutual gains, conflicting outcomes, or mutual losses) digital-HRM operates under, how it affects employee behavior and performance, and its implications holds significant theoretical and practical importance.

Reviewing past literature, the impact of digitalization and digital transformation on employee psychology and behavior has been extensively explored. Topics such as employee resilience [29], job satisfaction [30], engagement, well-being [31], and innovative behavior [32] have been investigated, providing insights into the symbiotic development between digitalization and employees. However, as a nascent topic, digital-HRM research has primarily focused on the implementation pathways of digital-HRM [33, 34] and its transformative outcomes at the organizational level [35]. For instance, there has been deep revision of HRM processes like employee recruitment, performance evaluation, and business training through digital technology [36–39] to support employees’ adaptation to digital disruptions [40], improve the productivity of HRM functions [36] and organizational effectiveness [37, 41, 42]. These studies offer valuable information for implementing digital-HRM within organizational contexts. Yet, in the current digital-HRM research, the exploration of its mechanisms impacting employee behavior at the individual level is still in its infancy. Only a few studies have begun to focus on employee-related factors such as fostering innovation [43] and enhancing performance [44]. However, there remains a significant gap in empirical exploration of the psychological mechanisms and processes underlying these effects. Especially in the post-pandemic era, employee mental health has received broader attention, particularly in exploring the predictive relationships between remote work, online work, and employees’ mental health and job performance [45–52]. Therefore, this study aims to explore whether digital-HRM affects employees’ psychological states through either mutual or conflicting outcomes, subsequently enhancing job performance, and the boundary conditions involved.

Digital-HRM, as a novel management approach emerging in the digital era, can, on one hand, achieve a comprehensive fusion of surreal, physical, and mental spaces, thereby cultivating a “ba” conducive to knowledge creation among employees [53]. “Ba” can be thought of as a shared space for emerging relationships. This space can be physical [e.g., office, dispersed business space], virtual [e.g., e-mail, teleconference], mental [e.g., shared experiences, ideas, ideals], or any combination of them [54]. Simultaneously, the features intrinsic to digital-HRM, including multiple co-creation, foreground thinking and value-added prediction, assume a paramount role in facilitating a transformative shift in employees’ cognitive processes and fostering an enduring spirit of innovation [53]. Therefore, this research opts to measure the impact brought by digital-HRM on employee innovative performance as the first outcome variable. On the other hand, while the digital era has prompted a transformation in employees’ work environments, evolving from traditional physical attributes to digital workplaces [40]. This transformation, while enhancing work efficiency and providing greater work convenience, has also increased the likelihood of employees engaging in non-work-related activities during work hours or at the workplace [55, 56], such as entertainment and browsing non-work-related websites [57–59], a phenomenon known as cyberloafing [60, 61]. However, digital-HRM transforms traditional HRM’s internal management into a participative style, granting employees more rights and freedom, thereby psychologically strengthening their sense of responsibility and ownership [8, 53]. Therefore, it may have a mitigating effect on cyberloafing. Consequently, we have chosen cyberloafing as the second outcome variable for this study. Based on the above analysis, we propose the following research questions:

Q1: How does digital-HRM influence employees’ psychological states and subsequently influences their innovation performance and cyberloafing?
Q2: What are the boundary conditions under which digital-HRM impacts employees’ psychological states and thereby influences their innovation performance and cyberloafing?

The job demands-resources theory posits that job resources encompass physiological, psychological, social, or organizational facets of work that stimulate positive work emotions and amplify employee engagement [62]. Simultaneously, these resources act as inhibitors of negative work emotions like burnout, which consequently mitigate slacking behavior [63]. Conversely, job demands necessitate consistent physical, cognitive, and emotional exertions, potentially entailing concomitant expenses such as work overload, interpersonal discord, and job insecurity [64]. Digital-HRM, emerging from the application and integration of digital technology, possesses the capacity not only for swift data supply and processing, aiding employees in problem exploration and real-time feedback for immediate enhancements, but also for harnessing algorithmic evolution, machine learning, and other data mining techniques to distill vital information amidst intricate contexts, this, in turn, fosters the enhancement of work efficiency [3, 40, 53]. Consequently, the current study contends that digital-HRM, as an emerging job resource in the digital era, with the potential to cultivate positive psychological experiences among employees, while simultaneously mitigating the emergence of adverse psychological states.

On the one hand, digital-HRM enhances employees’ productivity and cultivates their growth potential ‐ a hallmark characteristic of the concept of sense of work gain [65]. Considering that the notion of sense of work gain encapsulates the advantageous outcomes that employees attain amid the dynamics of organizational change, significantly influencing the organizational well-being [65, 66], we posits that digital-HRM proffers employees with sophisticated, efficacious services and robust organizational support. This, in turn, fortifies their competency and fosters favorable work dispositions, consequently augmenting their readiness and capacity for innovation. Therefore, digital-HRM holds the potential to advance employees’ innovative performance by bolstering their sense of work gain, a fundamental avenue through which the role of digital-HRM on employees’ psychology and behavior is first investigated within this study. On the other hand, distinguished by its capacity to foster workflow transparency and facilitate the visualization of work processes, digital-HRM engenders the reinforcement of employees’ perception regarding the transparency of HRM [40], thereby cultivating an environment of comparably equitable labor. This paradigm enables employees to gain lucidity regarding their contributions and performance, effectively dispelling potential uncertainties concerning HRM impartiality and managerial manipulation [53, 67]. As a result, the propensity for perceived inequity diminishes, consequently abating the sentiment of relative deprivation. In view of the potential utilization of cyberloafing as a compensatory mechanism to ameliorate deprivation [68–70], the current study advances the contention that relative deprivation constitutes a pivotal psychological mechanism to expound upon the adverse repercussions of digital-HRM on employees’ cyberloafing behavior. In light of this view, the current study posits that digital-HRM operates to mitigate employees’ engagement in cyberloafing by mitigating relative deprivation. This is the second mechanism by which this study reveals the impact of digital-HRM on employees’ psychology and behavior.

Moreover, given that digital technology forms the foundational underpinning for the implementation of digital-HRM, the degree of technological ease profoundly shapes the assimilation and execution of digital-HRM initiatives [3, 36]. Consequently, the current study aims to delve into the moderating role played by the perceived ease of technology use within the aforementioned operational mechanisms. It is noteworthy that prior scholarly investigations have substantiated the dual facets of technology’s impact in the workplace, encompassing both constraining and motivating dimensions [71]. In this context, the current study posits that perceived ease of technology use emerges as a pivotal boundary condition capable of influencing employees’ perceptions regarding the transformative implications of digital-HRM. Thus, this study seeks to scrutinize the moderating role assumed by perceived ease of technology use in delineating the nexus between digital-HRM and sense of work gain, as well as relative deprivation. Additionally, this study endeavors to empirically test the ensuing effects of moderated mediation.

The current study made several important contributions. Firstly, from a human-centered perspective and based on the HRM-WB-OIP theoretical model, we confirm that digital-HRM enhances employee performance through a mutual gains approach, marking a pioneering significance in the research of digital-HRM. Secondly, we integrate the job demands-resources theory into our research, exploring the impact of digital-HRM as a job resource on employee psychology and behavior, thereby enriching the application scope of the job demands-resources theory. Lastly, by examining the moderating role of perceived ease of technology use, we illustrate the boundary conditions of digital-HRM’s impact on employee psychology and behavior, also providing insights for enterprises on how to better implement the digital transformation of HRM, and comprehensively enhance the well-being and performance of employees and organizations.

## Theoretical background and hypotheses

### The job demands ‐ resources theory

The job demands -resources theory originated in the burnout literature and was first proposed by Demerouti et al. [62], and in its later development, the theory synthesized knowledge from various theories of work stress and job motivation and argued that burnout and work engagement are the result of various job characteristics [72]. The theory suggests that job characteristics can be categorized into job demands and job resources. The former relate to physical, psychological, social, or organizational aspects of work, which require sustained physical, cognitive, and emotional exertion, which in turn entails associated costs such as work overload, interpersonal conflict, and job insecurity, while the latter relate to elements of the same domains that have a motivational effect, such as coworker support, job autonomy, and performance feedback, which contribute to goal attainment and promote learning and personal growth [63, 64, 72]. Although both excessive job demands and scarce job resources can lead to burnout, reducing job demands alone will only alleviate burnout without affecting work engagement, only abundant job resources will trigger work engagement [72]. Therefore, by increasing job resources, organizations can increase employee engagement while attenuating burnout. In light of the above considerations, the current study aims to reveal the mechanism of action by which digital-HRM, as an emerging job resource in the digital era, promotes positive employee psychology and behavior while dampening negative reactions, and to further clarify how organizations can optimize the impact of digital-HRM in these areas.

### Linking digital-HRM to employee innovative performance via sense of work gain

Sense of work gain emanates from the construct of the “sense of gain”, which refers to a subjective feeling that individuals based on their perceived “objective acquisition” in the process of self-improvement and continuous advancement of their situation [73]. In this context, the objective acquisition includes not only material acquisition such as salary, but also spiritual acquisition such as organizational support and personal growth. Sense of work gain entails a comprehensive evaluation of the actual rewards and value that employees perceive in their work, specifically appraising the fairness and reasonableness of the relationship between their “work effort” and “work reward” [65]. This underscores the extent of contentment derived from individual needs fulfilled through work-related benefits [66]. Prior research has demonstrated that positive job characteristics and organizational support contribute to employees’ sense of work gain, thus fostering job well-being and engendering organizational citizenship behaviors [65, 74].

Based on the job demands-resources theory, job resources are considered to stimulate employees’ work motivation processes by satisfying their basic psychological needs, fostering engagement, and ultimately enhancing creativity and performance [72, 75–78]. Within this theoretical framework, digital-HRM plays a crucial role as a job resource, significantly fostering positive psychological experiences and thereby boosting employees’ innovative performance. We posit that digital-HRM serves as a job resource in three main areas: transforming the work environment, optimizing HRM processes, and enhancing employee services [40]. Firstly, digital-HRM transforms the work environment from a physical to a digital realm, not only enhancing work flexibility and connectivity but also creating an empathetic space that supports collaboration within the organization [40, 67, 79]. This shift meets employees’ basic psychological needs for flexibility and connectivity, promoting collaboration and innovation. Secondly, digital-HRM, through the refinement and intelligent transformation of management processes, has given rise to intelligent digital service systems and automated HR activities [1], which constitute new paradigms of governance and service. This not only improves work efficiency but also satisfies employees’ needs for skill development and personalized attention by providing timely feedback and customized services [53]. Furthermore, online workflows embedded in digital-HRM increase time efficiency, allowing HR professionals to divert from tedious daily tasks to focus on enhancing employee well-being and job satisfaction [40]. This shift gives employees more opportunities to develop skills and engage in meaningful activities [1], fulfilling their needs for achievement, self-efficacy, and self-actualization. In summary, we believe that digital-HRM, as a job resource, fully meets the diversified psychological needs of employees, providing comprehensive organizational support and significantly enhancing their sense of work gain.

Furthermore, we posit that the sense of work gain brought about by digital-HRM can further enhance employee innovative performance, which refers to the extent to which employees generate and implement new and useful ideas in the workplace [80]. Based on the aforementioned analysis, the sense of work gain derived from digital-HRM is primarily manifested on three levels: the foundational level of sense of work gain, which pertains to the fulfillment of work efficiency and income; the intermediate level of sense of work gain, which pertains to the fulfillment of respect; and the highest level of sense of work gain, which pertains to the fulfillment of self-actualization [66]. These varying levels of psychological fulfillment enable employees to have more flexible arrangements of work time and location [81, 82], concentrate on meaningful and valuable work [1], receive more personalized development [81, 83], and ensure that their contributions and value are duly recognized. This clarity in self-perception and growth leads to a greater willingness to learn new skills and knowledge [84, 85], as well as to share creativity and take risks within teams and the organization, thereby leading to improved innovation performance [3, 86]. Simultaneously, research results also indicate that a direct correlation between an elevated sense of work gain and favorable workplace encounters, coupled with heightened job contentment [65]. Significantly, individuals who manifest greater job satisfaction evince a proclivity for embracing more substantial risks and showcasing heightened creativity, this predisposition culminates in the manifestation of innovative behaviors and elevated innovative performance [33, 87]. Within this context, it is posited that digital-HRM can play a pivotal role in enhancing employees’ sense of work gain, thereby engendering an upsurge in their innovative performance. As a logical consequence, the following hypothesis is formulated:

H1: Sense of work gain mediates the positive relationship between digital-HRM and employee innovative performance. The higher the degree of digital-HRM, the more employee innovative performance is triggered by employees based on their sense of work gain.

### Linking digital-HRM to cyberloafing via relative deprivation

Relative deprivation refers to a negative subjective cognitive experience in which individuals or groups perceive themselves as disadvantaged or unfairly treated when making comparisons with other relevant groups [88]. Once individuals experience relative deprivation, it is easy to develop negative emotional as well as behavioral responses, and even trigger group behavior [89]. In terms of conceptual connotation, the meaning of relative deprivation is opposed to the meaning of sense of gain [73, 90]. Therefore, we believe that digital-HRM may diminish employees’ relative deprivation.

Similarly, according to the principles of the job demands-resources theory, job resources can alter the adverse perceptions and cognitions caused by job demands, thereby mitigating negative reactions during the evaluation process [72, 91–93]. Therefore, this study posits that digital-HRM, as an emerging job resource, can significantly change the adverse reactions caused by job demands on employees, such as feelings of relative deprivation. Firstly, digital-HRM increases the transparency of management activities through digital tools and platforms [53, 82], enabling employees to more easily access and understand decisions and policies related to their work [40]. This transparency directly alleviates the psychological stress and uncertainty caused by informational asymmetry, which are often major negative factors in traditional job demands. It helps reduce the likelihood of opaque operations or biased treatments, further eliminating concerns about improper interventions in HRM measures and alleviating feelings of relative deprivation. Meanwhile, as digital technology is progressively integrated within organizations, work processes are transforming towards higher transparency and visualization, steadily moving towards a “fully traceable” paradigm [3, 40], which grants unparalleled accessibility to information [67]. This enhances employees’ perceptions of fairness and justice, reducing their concerns about unfair treatment and the consequent feelings of relative deprivation. Secondly, digital-HRM alleviates many traditional job demands, such as repetitive tasks and time management pressures [1], through automation and intelligent technologies. Automation not only frees up time and energy for employees to engage in more creative and strategic work but also reduces the feeling of work overload [40], thereby decreasing the perception of deprivation. Overall, digital-HRM not only addresses the challenges brought by traditional job demands but also actively utilizes technological resources to optimize employees’ work experiences, thus weakening their feelings of relative deprivation.

We argue that this reduction in relative deprivation can attenuate employees’ cyberloafing, which is an IT way of idling on the job, and is often a consequence of perceived unfairness [60]. Relative deprivation, as a perception of unfair treatment, can trigger negative emotions that drive individuals to engage in inappropriate behaviors as a means to compensate for their perceived deprivation [94]. These behaviors are often aimed at quickly alleviating the sense of deprivation in the short term [95], such as addiction to online games [96] and theft [97]. Previous research has shown that cyberloafing can serve as a coping mechanism to alleviate feelings of exhaustion and emotional strain caused by workplace rejection or work-related stress [68, 69, 98].Therefore, we suggest that feelings of relative deprivation may drive employees to engage in cyberloafing as a means to cope with the emotional exhaustion. Thus, when digital-HRM reduces employees’ relative deprivation, we expect that their engagement in cyberloafing will also decrease. Combining the above analyses, we propose the following hypothesis:

H2: Relative deprivation mediates the negative relationship between digital-HRM and cyberloafing. The higher the degree of digital-HRM, the lower the amount of cyberloafing triggered by employees based on relative deprivation.

### The moderating effect of perceived ease of technology use

The conceivable applications and advantages of digital-HRM are contingent upon the degree to which employees embrace and integrate digital technologies within their work environment [3, 99, 100]. Consequently, our investigation delved into the moderating role played by the perceived ease of technology use. This construct pertains to the extent to which employees perceive the use of a specific technology as unproblematic and devoid of complexities [101].

Drawing from the self-determination theory, it is known that when individuals’ intrinsic needs for competence, autonomy, and relatedness are met, their self-motivation and well-being are enhanced [102]. In the context of the digital age, employees’ perceived ease of technology use becomes a key factor in fulfilling these psychological needs. Firstly, the perceived ease of technology use directly strengthens the sense of autonomy in their work. When technology is perceived as user-friendly, employees can complete tasks more quickly and efficiently, reducing delays and frustrations caused by technological barriers [103]. Moreover, as the ease of using technology increases, employees feel more control and freedom of choice when using technology [104]. This enhanced autonomy encourages them to adopt and utilize technology more proactively, rather than feeling forced to adapt, thus improving job satisfaction and engagement [105–107]. Secondly, a high perception of technology’ ease of use enhances their sense of competence. When employees believe technology is easy to master and use, they are more likely to feel competent at their jobs and able to complete tasks more quickly and receive positive feedback [108, 109], thereby boosting their sense of self-efficacy and accomplishment. Indeed, using a longitudinal design, Xanthopoulou et al. [110] discovered that individuals with high self-efficacy reported higher levels of autonomy and growth opportunities over time, subsequently influencing their experiences of self-motivation and well-being. In a technology-driven work environment, a smooth technological user experience is a crucial factor in enhancing employees’ sense of competence. Lastly, the perceived ease of technology use lowers the barriers to communication and collaboration, not only enabling employees to interact more effectively with colleagues and establish and maintain positive work relationships but also making them feel more interconnected and supported [111], satisfying their social belonging and relatedness needs. It also promotes collaboration and information sharing among team members [112], contributing to improved overall team efficiency and innovative capacity. Effective participation and contribution within the team enhance employees’ sense of social value, reducing feelings of marginalization or relative deprivation.

In conclusion, the perceived ease of technology use is crucial for satisfying employees’ needs for autonomy, competence, and relatedness. The higher the employees’ perceived ease of technology use, the more likely they are to experience enhanced job satisfaction, engagement, well-being, and team support. Therefore, we believe that employees with a higher perceived ease of technology use are more likely to experience increased sense of work gain and reduced feelings of relative deprivation. Building upon the aforementioned arguments, we propose the following moderating hypotheses:

H3a: Perceived ease of technology use moderates the positive relationships between digital-HRM and sense of work gain, such that the relationships are stronger when perceived ease of technology use is higher rather than lower.
H3b: Perceived ease of technology use moderates the negative relationships between digital-HRM and relative deprivation, such that the relationships are stronger when perceived ease of technology use is higher rather than lower.

Combining H1, H2, H3a and H3b, this study further proposes two moderated mediation hypotheses. Specifically, for employees with a high-level perceived ease of technology use, they will be more inclined to embrace digital-HRM, from which they will experience more need satisfaction and thus higher sense of work gain, which will motivate them to exhibit higher innovative performance; at the same time, employees’ perception of relative deprivation will be further weakened, which will further inhibit them from committing cyberloafing behaviours.

H4a: The positive indirect relationship between digital-HRM and employee innovative performance through sense of work gain will be moderated by perceived ease of technology use such that the relationship will be stronger (vs. weaker) when perceived ease of technology use is higher (vs. lower).
H4b: The negative indirect relationship between digital-HRM and cyberloafing through relative deprivation will be moderated by perceived ease of technology use such that the relationship will be stronger (vs. weaker) when perceived ease of technology use is higher (vs. lower).

**Fig 1:**
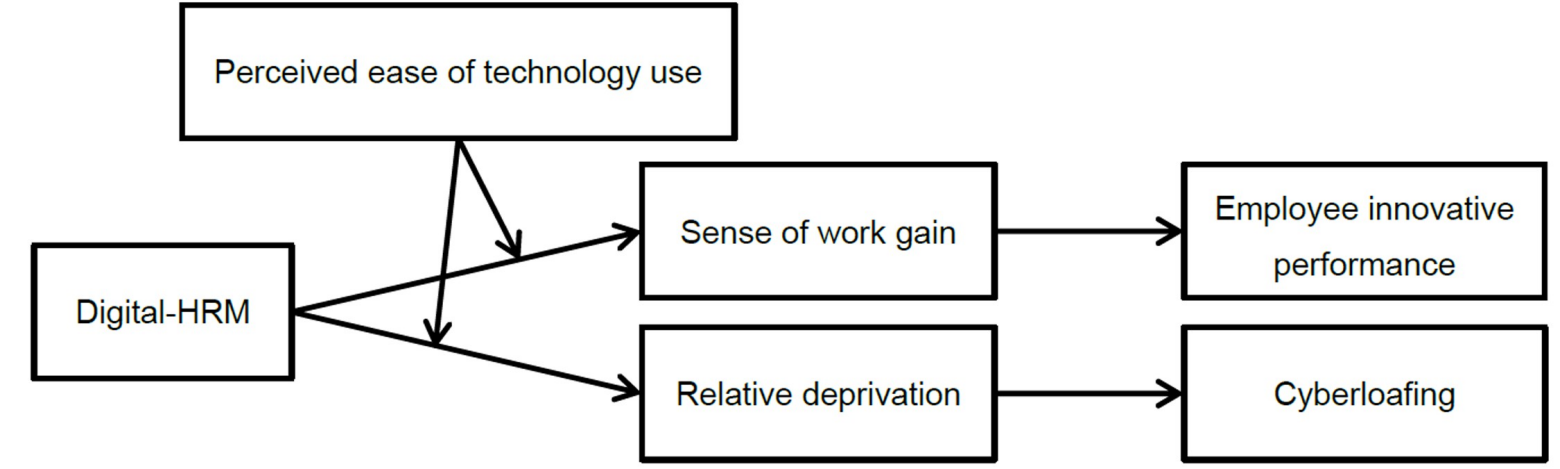
Theoretical framework.

The research model of this paper is shown in Fig 1.

## Methodology

### Sample and data collection

This study develops a moderated mediation model that proposes the positive effects of digital-HRM, an emerging job resource, on employee innovative performance as well as the negative effects of cyberloafing through the dual mediation of sense of work gain and relative deprivation and the moderating effects of perceived ease of technology use. To this end, we surveyed full-time employees from various industries in China (including Internet, finance, energy, construction, and retail) through the Credamo. Credamo is a large-scale intelligent online research platform which allows the distribution of questionnaires to those who meet the inclusion criteria.

Our study started on December 7, 2022 and ended on December 22, 2022. We used a questionnaire to distribute 550 questionnaires on Credamo, offering a 5¥ incentive per questionnaire. Each questionnaire included a statement informing participants of the nature, confidentiality, and objectives of the study. Since we utilized an online distribution method for the questionnaires, all participants had the right to decide whether to participate, ensuring voluntary involvement. Once participants completed the questionnaire and submitted it, they indicated their consent to participate in our study. The Academic Committee of the Research Institute of International Economics and Management, Xihua University, exempted this study from ethical review and since our study ensured the willingness of the participants to participate in the survey, the Academic Committee stated that this study did not require any written or verbal formal consent from the participants. To ensure the quality of the sample, we performed some operations before we started collecting data. Firstly, we restricted the survey population before distributing the questionnaires, and only allowed the employees of the enterprises to fill in the questionnaires; secondly, we restricted the IP addresses when distributing the questionnaires, and prohibited the same IP address from answering the questionnaires more than once; and at the same time, we also restricted the answer range, and only allowed one IP address to answer the questionnaires within 5km, so that we could avoid the situation that the group answering would interfere with the data results. Finally, we also set up interference questions to exclude the questionnaires that were not filled out carefully to further ensure the quality of the questionnaire. Moreover, to avoid common method variance [113], we conducted the survey at three points in time. At time 1, we distributed 550 questionnaires, including questions regarding digital-HRM, perceived ease of technology use, and control variables. And, 548 responses were received. At time 2, after a gap of one week, questionnaires on sense of work gain and relative deprivation were sent to the 548 initial participants. And, 523 responses were collected, generating a response rate of 95.44%. At time 3, one week after time 2, questionnaires on employee innovative performance and cyberloafing were sent to the 523 participants, 502 responses were collected. After excluding incomplete responses as well as straight-line responses and outliers, 487 valid questionnaires were obtained, with an effective response rate of 88.55%. Data are available in S1 Data.

Due to the unavailability of official data regarding the characteristics of employees in the context of digital-HRM, we refer to the study by Martin et al. [114] and choose the data of the entire employed population of China at the end of 2022 as the reference data in our study to test the representativeness of the sample. The reference data is sourced from the China Population and Employment Statistical Yearbook 2023 (https://www.zgtjnj.org/navibooklist-n3024011706-1.html). It is worth noting that, comparing the profile of the respondents with that of the entire working population in China profile, we find that the sample is slightly more educated, with a higher percentage of individuals with bachelor’s degrees compared to the entire employed population of China; furthermore, the age distribution of the sample is more youthful, with the majority of the group under 45 years of age, while the percentage of the group over 50 years of age is significantly lower than the percentage of the national employed population. This result suggests that our respondents are generally more educated and more skewed toward a younger age group. In light of the fact that millennial workforce has become the largest group in the labor market at the present time [115], and also given precedents set by related research [116–118], we believe that such a discrepancy is reasonable and consistent with the topic of our current study.

### Measurements

Except for the measurement of the digital-HRM variable, which was measured using a self-developed scale, the scales selected for the measurement of the remaining variables in this paper are more mature scales that have demonstrated high reliability and validity in previous studies. We used a five-point Likert scale to measure all variables from “strongly disagree” (1) to “strongly agree” (5).

For the current study, digital-HRM was measured by 6-items scale that we developed ourselves, example items included “My organization uses digital technology to analyze and make decisions on assessment-related data.” Perceived ease of technology use was measured through Davis’s 4-item scale [101], example items included “Learning to operate the digital technology is easy for me.” Relative deprivation was measured using 4-items scale adapted from Cho et al. [119], example items included “When I compare what I have with my colleagues, I feel like I’m missing something.” Sense of work gain was measured using 8-items scale adapted from Zhu and Liu [65], example items included “Work has allowed me to become proficient in job knowledge and skills.” Cyberloafing was measured using 6-items scale adapted from Blau et al. [120], these items included “Receiving non-work related emails during working hours.” Employee innovative performance was measured using 6-items scale developed from Janssen and Van Yperen [121], these items included “I offer new ideas to improve existing working conditions.” Specific measurement items for each variable can be found in S1 Appendix.

Additionally, we included five control variables, namely age, gender, education, work experience and firm ownership.

### Techniques for data analysis

The study uses the partial least squares structural equation modeling (PLS-SEM) technique. The SmartPLS software was employed. To test the moderated mediation effect (H4a and H4b), the PROCESS macro suggested by Hayes [122] was used. For the moderated mediation was tested by using Model 7.

## Results and data analysis

### Demographic profile of participants

The current study implemented the survey approach to collect data from respondents of various age brackets, gender, education, work experience, and firm ownership. The data collected from the respondents depicted 44.4% of the individuals as males and 55.6% as females. In terms of age bracket, 39.6% of the individuals were aged between 20–30 years, 54% were aged between 31–45 years, 5.8% were aged between 46–55 years, and 0.6% were aged 56 years and above. In terms of education level, 3.1% of the respondents are in high school or technical secondary school and below, 11.3% have a junior college degree, 69.2% have an undergraduate degree, and 16.4% have a graduate degree or above. In terms of work experience, 56.1% of all respondents have 4–10 years of work experience, while 27.5% have only three years or less, and 16.4% have more than ten years of work experience. In terms of firm ownership, 29.6% of the respondents were in state-owned enterprise, 62% were in private Chinese enterprise, 6.8% were in wholly foreign-owned enterprise, and 1.6% were in other ownerships, such as joint ventures (See Table 1).

**Table 1:** Demographics characteristics of respondents.

Variables	Description	Frequency	Percentage %	Total
Age	20–30 years	193	39.6	487
	31–45 years	263	54	
	46–55 years	28	5.8	
	56 & above	3	0.6	
Gender	Male	216	44.4	487
	Female	271	55.6	
Education	High school or technical secondary school and below	15	3.1	487
	Junior college	55	11.3	
	Undergraduate	337	69.2	
	Graduate and above	80	16.4	
Work experience	1–3 years	134	27.5	487
	4–10 years	273	56.1	
	Above years	80	16.4	
Firm ownership	state-owned enterprise	144	29.6	487
	private Chinese enterprise	302	62	
	wholly foreign-owned enterprise	33	6.8	
	others	8	1.6	

Source: Developed by authors on the basis of collected data.

### Descriptive statistics and correlations

Tables 2 and 3 shows the means and standard deviations of and the correlations among the study variables. All of control variables are related to our main variables respectively, so they were controlled for our main variables in the subsequent analysis. The results of the correlation analysis provide preliminary support for our hypotheses.

**Table 2:** Descriptive statistics.

Variable	Mean	SD	Minimum	Maximum	25% Quantile	Median	75% Quantile
Age	32.82	6.674	20	63	28	32	36
Gender	0.44	0.497	0	1	0	0	1
Education	2.99	0.633	1	4	3	3	3
Work experience	6.69	4.669	1	30	3	6	9
Firm ownership	1.80	0.626	1	4	1	2	2
DHRM	4.174	0.591	1.67	5	4	4.333	4.5
PEUT	4.167	0.518	2.25	5	4	4.25	4.5
SWG	4.064	0.606	1.88	5	3.75	4.25	4.5
RD	2.365	0.745	1	4.75	1.75	2.25	2.75
EIP	3.674	0.764	1.33	5	3.167	3.833	4.333
Cyberloafing	1.987	0.580	1	4.83	1.5	1.833	2.333

Notes: n = 487

*p<0.05

**p<0.01

Gender:1 = Male; 0 = Female

Education: 1 = high school or technical secondary school and below; 2 = junior college; 3 = undergraduate; 4 = graduate and above

Firm ownership: 1 = state-owned enterprise; 2 = private Chinese enterprise; 3 = wholly foreign-owned enterprise; 4 = others

DHRM = digital-HRM

PETU = perceived ease of technology use

SWG = sense of work gain

RD = relative deprivation

EIP = employee innovative performance

Source: Developed by authors on the basis of collected data.

**Table 3:** Correlations.

	Variable	1	2	3	4	5	6	7	8	9	10	11
1	Age	—										
2	Gender	0.101*	—									
3	Education	-0.068	-0.116*	—								
4	Work experience	0.711 **	0.131**	-0.045	—							
5	Firm ownership	-0.067	0.001	-0.057	-0.180**	—						
6	DHRM	0.070	0.123**	0.024	0.144**	-0.054	[0.814]					
7	PEUT	0.030	0.066	0.128**	0.064	-0.028	0.452**	[0.727]				
8	SWG	0.113*	0.111*	0.065	0.182**	-0.030	0.637**	0.479**	[0.858]			
9	RD	-0.99*	-0.047	-0.115*	-0.151**	0.008	-0.420**	-0.444**	-0.658**	[0.727]		
10	EIP	0.111*	0.132**	0.175**	0.198**	-0.102*	0.576**	0.555**	0.623**	-0.518**	[0.894]	
11	Cyberloafing	-0.206**	-0.067	-0.015	-0.267**	0.132**	-0.371**	-0.264**	-0.421**	0.366**	-0.415**	[0.815]

DHRM = digital-HRM

PETU = perceived ease of technology use

SWG = sense of work gain

RD = relative deprivation

EIP = employee innovative performance

Source: Developed by authors on the basis of collected data.

### Assessment of common method bias

Before assessing the research model, we first assessed the common method bias using the full collinearity test. The result showed that the variance inflation factor (VIF) values of all constructs ranged between 1.150 and 2.582 (below 3.33; [123]). In addition, we adopted Harman’s single factor analysis to test common method variance [124]. The results show that the first principal component explained only 32.670% of the variance variation, which was less than the 40% threshold. Thus, we concluded that there is no serious homology variance problem in the current research.

### Assessment of measurement model

**Fig 2:**
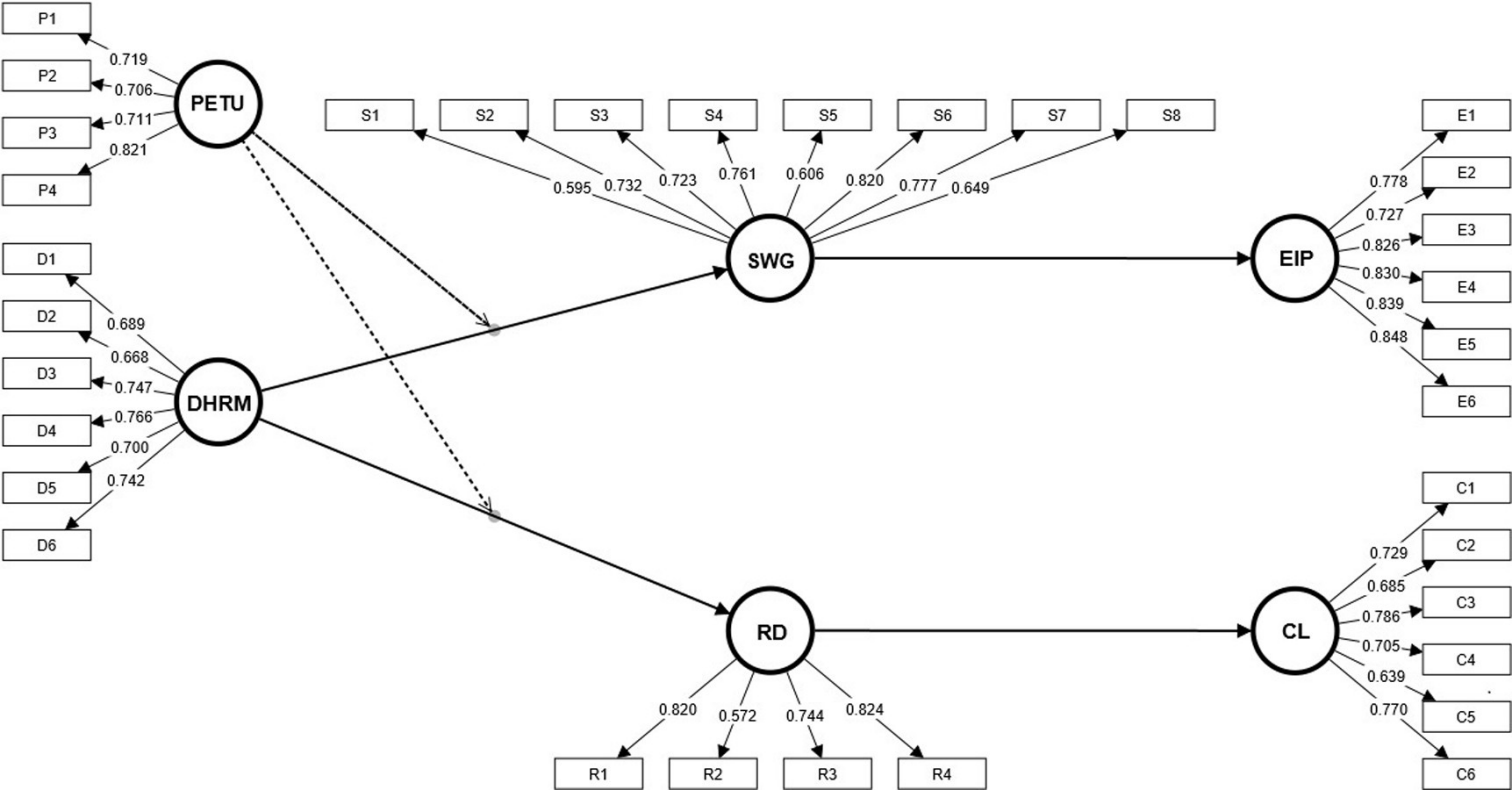
Factor loadings.

It was verified that all reflective constructs presented a Cronbach’s alpha (α) and composite reliability (CR) greater than 0.7 [125]. Thus, construct reliability is adequate. Convergent validity was checked against average variance extracted (AVE). As reported in Table 4, the latent variables reach convergent validity because their AVE measurements exceed 0.5 [126]. Also, as shown in Fig 2, some factor loadings were below 0.7 but higher than 0.5, according to Hair Jr et al. [127], an item with outer loading >0.50 should not be removed if the construct achieved satisfactory values for AVE (>0.50) and internal consistency (>0.70), thus confirming reliability of the constructs.

**Table 4:** Measurement model: Discriminant validity.

Latent variable				HTMT Ratios					
	α	CR	AVE	1	2	3	4	5	6
Cyberloafing	0.815	0.826	0.519						
DHRM	0.814	0.818	0.518	0.456					
EIP	0.894	0.901	0.655	0.483	0.675				
PETU	0.727	0.736	0.549	0.343	0.587	0.683			
RD	0.727	0.747	0.558	0.475	0.548	0.633	0.605		
SWG	0.858	0.867	0.507	0.501	0.764	0.708	0.611	0.821	

Notes: DHRM = digital-HRM

PETU = perceived ease of technology use

SWG = sense of work gain

RD = relative deprivation

EIP = employee innovative performance

PETU = perceived ease of technology use

Source: Developed by the authors on the basis of collected data using Smart PLS.

Discriminant validity was checked with the Heterotrait-Monotrait (HTMT) ratio correlation [128]. Based on Table 4, all the HTMT values are lower than the conservative threshold value of 0.85, indicating discriminant validity is established between all the constructs. Overall, the model provides no reliability or validity concerns. In addition, model fit was assessed using root mean square residual (SRMR), a SRMR value of 0.054 showed an acceptable model fit.

### Assessment of structural model

In the structural model evaluation (Table 5), the criteria to be considered include the assessment of possible collinearity problems through the determination coefficient (R^2^) and the blindfolding-based cross-validated redundancy measure (Q^2^). As can be seen in Table 4, R^2^ for cyberloafing = 0.197, R^2^ for employee innovative performance = 0.430, R^2^ for relative deprivation = 0.292, R^2^ for sense of work gain = 0.495. The values for the Stone-Geisser Q^2^ statistic presented for the four endogenous constructs are above zero. In consequence, the structural model in this study was acceptable.

**Table 5:** Structural model: Evaluation indicators.

Factor	Cross-Validated Redundancy		Coefficient of Determination		
	SSO	SSE	Q^2^ [= 1–SSE/SSO]	R^2^	Adjusted R^2^
Cyberloafing	2922.000	2639.930	0.097	0.197	0.187
EIP	2922.000	2125.159	0.273	0.430	0.423
RD	1948.000	1654.871	0.150	0.292	0.280
SWG	3896.000	2942.605	0.245	0.495	0.487

Notes: SWG = sense of work gain

RD = relative deprivation

EIP = employee innovative performance

Source: Developed by the authors on the basis of collected data using Smart PLS.

### Hypothesis testing

**Fig 3:**
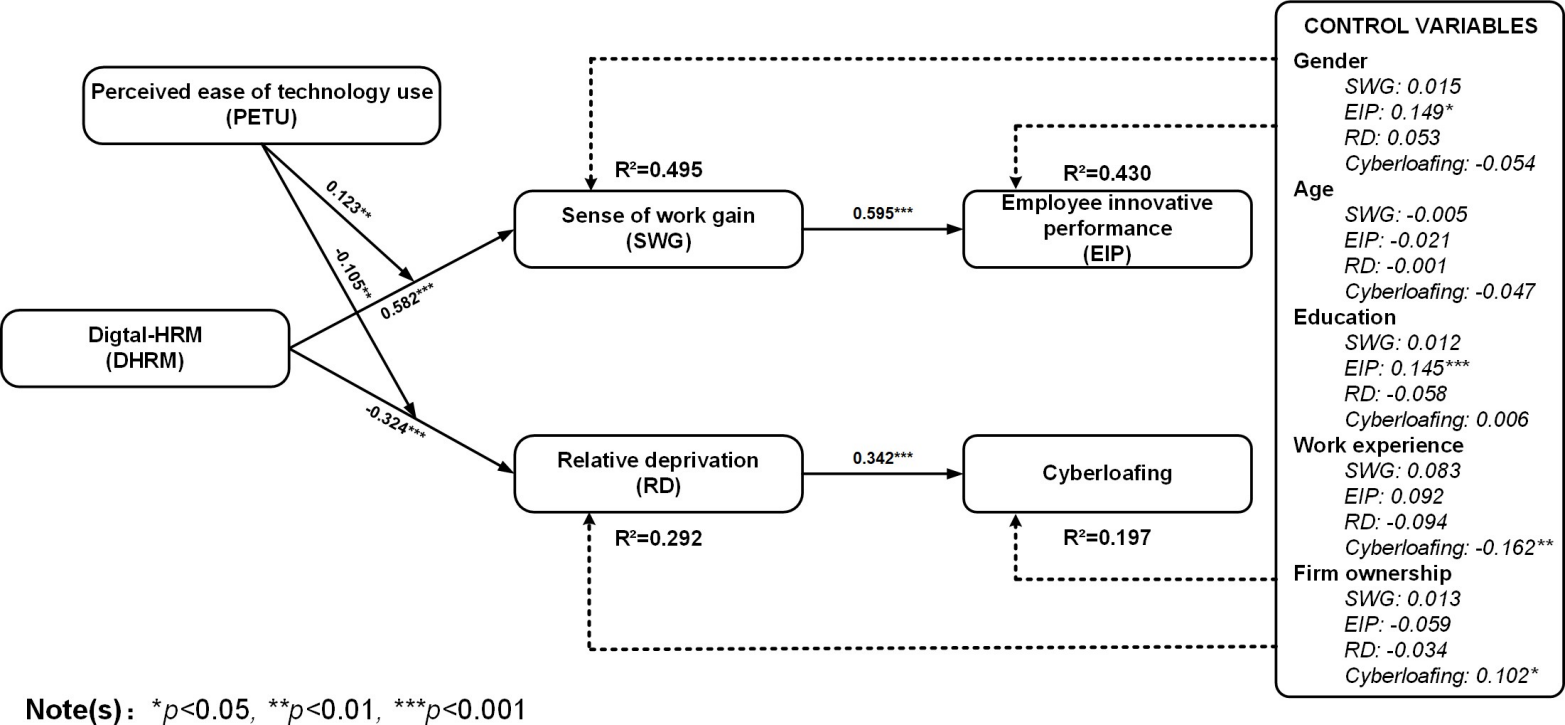
Results of partial least square SEM model.

Path analysis with a 5000-sample bootstrapping run was performed to test the proposed hypotheses. Analysis results are shown in Table 6 and Fig 3.

**Table 6:** Results of hypothesis testing.

Hypotheses	Path	β	SD	t-value	Result
H1	DHRM→SWG→EIP	0.346	0.036	9.641***	Supported
H2	DHRM→RD→Cyberloafing	-0.111	0.022	4.921***	Supported
H3a	PETU*DHRM→SWG	0.123	0.039	3.197**	Supported
H3b	PETU*DHRM→RD	-0.105	0.035	2.992**	Supported
H4a	PETU*DHRM→SWG→EIP	0.073	0.023	3.125**	Supported
H4b	PETU*DHRM→RD→Cyberloafing	-0.036	0.014	2.661**	Supported

Notes: n = 487

*p<0.05

**p<0.01

***p<0.001.

DHRM = digital-HRM

PETU = perceived ease of technology use

SWG = sense of work gain

RD = relative deprivation

EIP = employee innovative performance

Source: Developed by the authors on the basis of collected data using Smart PLS.

Firstly, digital-HRM was positively associated with sense of work gain (β = 0.582, p < 0.001), with the latter also positively influenced employee innovative performance (β = 0.595, p < 0.001). The indirect effect of digital-HRM on employee innovative performance through sense of work gain was also significant (β = 0.346, p < 0.001), providing support for H1. Similarly, digital-HRM was negatively associated with relative deprivation (β = -0.324, p < 0.001), with the latter positively influenced cyberloafing (β = 0.342, p < 0.001). The indirect effect of digital-HRM on cyberloafing through relative deprivation was also significant (β = -0.111, p < 0.001), providing support for H2.

**Fig 4:**
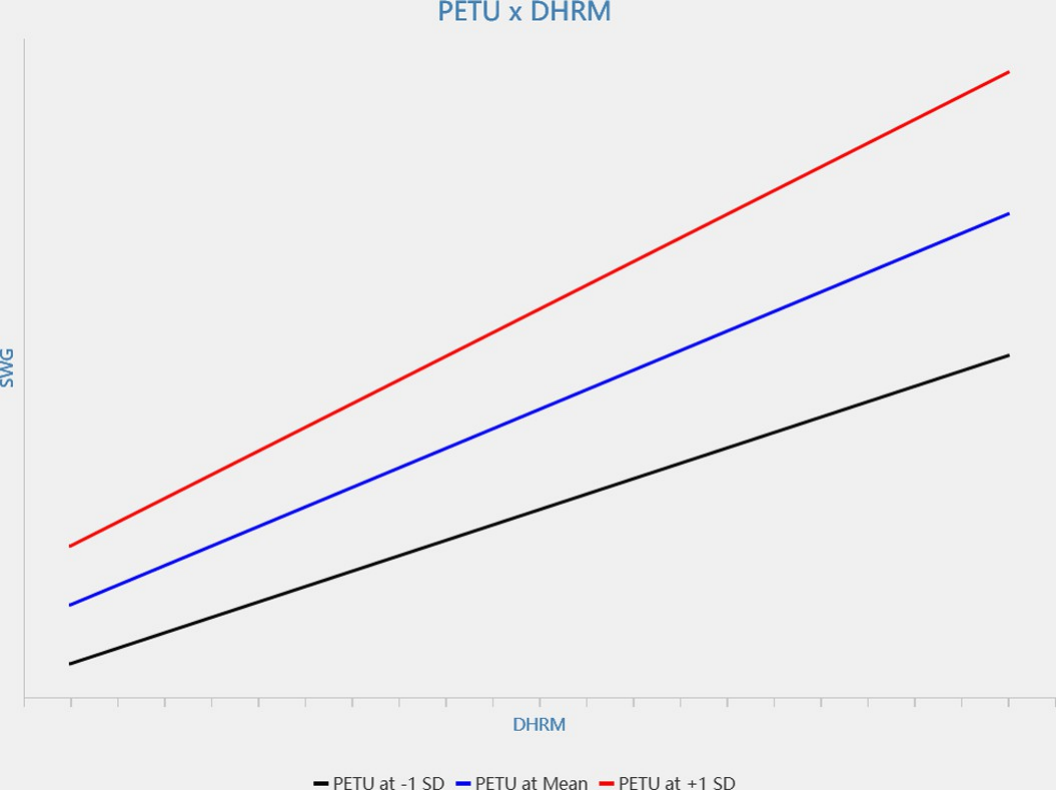
The interactive effect of digital-HRM and perceived ease of technology use on sense of work gain.

**Fig 5:**
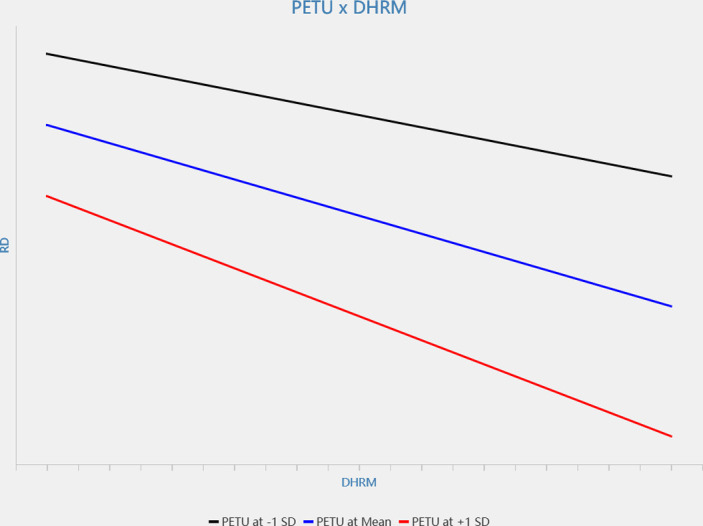
The interactive effect of digital-HRM and perceived ease of technology use on relative deprivation.

Secondly, results showed that perceived ease of technology use moderated the relationship between digital-HRM and sense of work gain, indicated by regression coefficient the interaction term on sense of work gain (β = 0.123, p < 0.01, see Fig 3) in that the relationship is stronger when perceived ease of technology use is high (see Fig 4). At the same time, perceived ease of technology use also moderated the relationship between digital-HRM and relative deprivation, indicated by regression coefficient the interaction term on relative deprivation (β = -0.105, p < 0.01, see Fig 3) in that the relationship is stronger when perceived ease of technology use is high (see Fig 5). Hence, H3a and H3b are verified.

Finally, in order to test the moderated mediation effects (H4a and H4b), we used PROCESS technique. Results showed that perceived ease of technology use moderated the relationship between digital-HRM and employee innovative performance via sense of work gain (conditional indirect effect (+1 SD) = 0.3678, bootSE = 0.0628, 95% confidence interval (CI) [0.2521,0.4983]; conditional indirect effect (M) = 0.3011, bootSE = 0.0477, 95% CI [0.2133,0.4012]; conditional indirect effect (- 1 SD) = 0.2344, bootSE = 0.0396, 95% CI [0.1637,0.3192]), the 95% CI in the difference of these effects excluded zero (difference = 0.1333, 95% CI [0.0466, 0.2197]). Overall, H4a is supported.

Perceived ease of technology use also moderated the relationship between digital-HRM and cyberloafing via relative deprivation (conditional indirect effect (+1 SD) = -0.0969, bootSE = 0.0245, 95% CI [-0.1518,-0.0545]; conditional indirect effect (M) = -0.0735, bootSE = 0.0176, 95% CI [-0.1126,-0.0438]; conditional indirect effect (- 1 SD) = -0.0502, bootSE = 0.0144, 95% CI [-0.0817,-0.0259]), the 95% CI in the difference of these effects excluded zero (difference = -0.0467, 95% CI [-0.0872, -0.0132]). Overall, H4b is supported.

### Robustness test

To further test the hypothesis results, we conducted a series of robustness tests.

First, we reanalyzed the data after removing all control variables. Using SmartPLS for analysis, the results, as shown in Table 7, still support the proposed hypothesis. Also, using PROCESS to test the moderated mediation effect, the results indicate that when the perceived ease of technology use is high, the indirect effect of sense of work gain is 0.4044, with a 95% CI [0.2816, 0.5359]; when the perceived ease of technology use is low, the indirect effect of sense of work gain is 0.2534, with a 95% CI [0.1809, 0.3382]. The difference in indirect effects between high and low conditions is 0.1510, with a 95% CI [0.0609, 0.2457], excluding 0, supporting H4a. When the perceived ease of technology use is high, the indirect effect of relative deprivation is -0.1094, with a 95% CI [-0.1675, -0.0642]; when perceived ease of technology use is low, the indirect effect of relative deprivation is -0.0539, with a 95% CI [-0.0862, -0.0282]. The difference in indirect effects between the high and low conditions is -0.0555, with a 95% CI [-0.1005, -0.0181], excluding 0, supporting H4b.

**Table 7:** Results of hypothesis testing.

Hypotheses	Path	β	SD	t-value	Result
H1	DHRM→SWG→EIP	0.375	0.037	10.275***	Supported
H2	DHRM→RD→Cyberloafing	-0.125	0.024	5.245***	Supported
H3a	PETU*DHRM→SWG	0.131	0.039	3.326**	Supported
H3b	PETU*DHRM→RD	-0.115	0.036	3.212**	Supported
H4a	PETU*DHRM→SWG→EIP	0.082	0.025	3.265**	Supported
H4b	PETU*DHRM→RD→Cyberloafing	-0.043	0.015	2.864**	Supported

Notes: n = 487

*p<0.05

**p<0.01

***p<0.001.

DHRM = digital-HRM

PETU = perceived ease of technology use

SWG = sense of work gain

RD = relative deprivation

EIP = employee innovative performance

Source: Developed by the authors on the basis of collected data using Smart PLS.

Secondly, we performed a second test of the mediation and moderation hypotheses using hierarchical linear regression via SPSS software. As shown in Tables 8 and 9, the maximum variance inflation factor (VIF) coefficient of all models was 2.157, which was less than the threshold of 5 [123, 129], so no serious multicollinearity was found. Moreover, the F value of all models reached the level of significance (p <0.01), indicating that the model was statistically significant.

**Table 8:** Results of hierarchical linear regression analysis.

	SWG			EIP			
	M1	M2	M3	M4	M5	M6	M7
Age	-0.031	0.007	0.004	-0.041	-0.008	-0.023	-0.011
Gender	0.098*	0.029	0.012	0.131**	0.071	0.074*	0.059
Education	0.083	0.059	0.015	0.193***	0.172***	0.144***	0.148***
Work experience	0.196**	0.090	0.078	0.209**	0.116*	0.093	0.079
Firm ownership	0.008	0.023	0.015	-0.056	-0.042	-0.061	-0.052
DHRM		0.620***	0.575***		0.545***		0.294***
SWG						0.589***	0.405***
PETU			0.300***				
DHRM×PETU			0.182***				
F	4.882***	57.579***	56.101***	9.932***	48.941***	58.963***	61.925***
R^2^	0.048	0.419	0.484	0.094	0.380	0.424	0.475
Adj R^2^	0.038	0.411	0.476	0.084	0.372	0.417	0.467
D-W	\	1.953	1.955	\	2.005	1.937	2.007
Max VIF	2.113	2.143	2.151	2.113	2.143	2.153	2.157

Notes: n = 487

*p<0.05

**p<0.01

***p<0.001.

DHRM = digital-HRM

PETU = perceived ease of technology use

SWG = sense of work gain

EIP = employee innovative performance

Source: Developed by the authors on the basis of collected data using Smart PLS.

**Table 9:** Results of hierarchical linear regression analysis.

	RD			Cyberloafing			
	M8	M9	M10	M11	M12	M13	M14
Age	0.012	-0.012	-0.010	-0.044	-0.064	-0.048	-0.061
Gender	-0.042	0.003	0.020	-0.037	0.000	-0.023	0.000
Education	-0.128**	-0.112**	-0.063	-0.027	-0.014	0.016	0.013
Work experience	-0.165*	-0.096	-0.086	-0.217**	-0.159**	-0.161**	-0.136*
Firm ownership	-0.028	-0.038	-0.031	0.088*	0.080	0.098*	0.089*
DHRM		-0.405***	-0.315***		-0.339***		-0.243***
RD						0.337***	0.238***
PETU			-0.359***				
DHRM×PETU			-0.143**				
F	4.035**	19.818***	24.016***	8.532***	19.044***	18.791***	21.327***
R^2^	0.040	0.199	0.287	0.081	0.192	0.190	0.238
Adj R^2^	0.030	0.189	0.275	0.072	0.182	0.180	0.226
D-W	\	2.071	2.040	\	2.029	1.946	2.017
Max VIF	2.113	2.143	2.151	2.113	2.143	2.142	2.155

Notes: n = 487

*p<0.05

**p<0.01

***p<0.001.

DHRM = digital-HRM

PETU = perceived ease of technology use

RD = relative deprivation

Source: Developed by the authors on the basis of collected data using Smart PLS.

According to Baron and Kenny [130] and Liu and Zhang [131], we utilized the three-step regression method to test whether SWG and RD has a mediating effect. Step 1: DHRM (M5/M12) has a direct effect on EIP/CL. Step 2: DHRM (M2/M9) has a direct effect on SWG/RD. Step 3: After SWG/RD is introduced, the effect of DHRM on EIP/CL still exists. As shown in M7/M14, p < 0.001 with statistical significance. With DHRM, the β coefficient dropped/rised to 0.294/ -0.243, indicating that SWG/RD plays a positive/negative partial mediates effect relationship between DHRM and EIP. To sum up, H1 and H2 was supported.

From M3 in Table 8, when sense of work gain is used as the dependent variable, perceived ease of technology use and the interaction term between digital-HRM and perceived ease of technology use are added to the basis of M2. The results show that perceived ease of technology use positively moderates the relationship between digital-HRM and sense of work gain (β_DHRM_ = 0.575,p<0.001; β_DHRM×PETU_ = 0.182, p<0.001), thus confirming H3a. Similarly, from M14 in Table 9, when relative deprivation is the dependent variable, perceived ease of technology use and the interaction term between digital-HRM and perceived ease of technology use are added to the basis of M9. It is found that perceived ease of technology use positively moderates the relationship between digital-HRM and relative deprivation(β_DHRM_ = -0.243,p<0.001; β_DHRM×PETU_ = -0.143, p<0.01), thus confirming H3b.

Finally, we use the PROCESS macro model and the Bootstrap to retest the mediation effects of sense of work gain and relative deprivation and the moderation effect of perceived ease of technology use for robustness, setting the sample size to 5000. If the 95% confidence interval does not include 0, it indicates that the results are significant and the hypothesis is further confirmed. For the robustness test of the mediation effects, as shown in Table 10, the indirect effect of sense of work gain has a 95% CI [0.2306, 0.4269], not including 0, suggesting that sense of work gain significantly mediates between digital-HRM and employee innovative performance. The indirect effect of relative deprivation has a 95% CI [-0.1367, -0.0575], not including 0, indicating that relative deprivation significantly mediates between digital-HRM and cyberloafing, further supporting the original hypothesis. As for the robustness test of the moderation effect, the results indicate that perceived ease of technology use significantly moderates between digital-HRM and sense of work gain(β = 0.2521, p < 0.001); it also significantly moderates between digital-HRM and relative deprivation(β = -0.2438, p < 0.01), as shown in Table 10. Under conditions of high perceived ease of technology use, the positive/negative impact of digital-HRM on sense of work gain/relative deprivation is enhanced, thereby further supporting the original hypotheses.

**Table 10:** Results of mediation effect and moderated mediation effect.

		Effect	Boot SD	95% Confidence Interval
Mediation path				
DHRM→SWG→EIP	Direct effect	0.3795	0.0558	[0.2699,0.4891]
	Indirect effect	0.3247	0.0496	[0.2306,0.4269]
	Total effect	0.7042	0.0473	[0.6112,0.7972]
DHRM→RD→Cyberloafing	Direct effect	-0.2382	0.0436	[-0.3240,-0.1524]
	Indirect effect	-0.0946	0.0201	[-0.1367,-0.0575]
	Total effect	-0.3328	0.0410	[-0.4134,-0.2522]
Moderate path				
DHRM*PETU→SWG	PETU(+1SD)	0.7202	0.0572	[0.6077,0.8326]
	PETU(M)	0.5896	0.0407	[0.5097,0.6695]
	PETU(-1SD)	0.4591	0.0412	[0.3781,0.5400]
DHRM*PETU→RD	PETU(+1SD)	-0.5237	0.0828	[-0.6863,-0.3611]
	PETU(M)	-0.3974	0.0588	[-0.5130,-0.2819]
	PETU(-1SD)	-0.2712	0.0596	[-0.3883,-0.1541]

Notes: n = 487

DHRM = digital-HRM

SWG = sense of work gain

RD = relative deprivation

EIP = employee innovative performance

Source: Developed by the authors on the basis of collected data using Smart PLS.

Although this study utilized a three-stage time-lagged method to collect data, it could not assert causality between variables. Therefore, building on the original measurement tools and data, we conducted a second follow-up survey of 487 respondents from November 24, 2023 to November 30, 2023, using the same questionnaire as the first survey. A total of 248 questionnaires were collected. We used IP addresses to match the questionnaires from the two collections, and after eliminating incomplete and uniform responses, a total of 241 valid questionnaires were obtained.

Before testing, we used the χ^2^, CFI, TLI, and RMSEA to evaluate the model’s fit. Compared to the χ^2^ value, the other fit indices are less influenced by the sample size and account for both the goodness of fit and the model’s complexity. For the model’s fit, χ^2^ = 203.093, CFI = 0.946, TLI = 0.907, and RMSEA = 0.064, all of which are at satisfactory levels.

The structural coefficients estimated are presented in Table 11. According to the analysis, when controlling for stability effects (i.e., the influence of variables measured at Time 1 on the same variables measured at Time 2), Time 1’s Digital-HRM still significantly affects Time 2’s sense of work gain(β = 0.191, p<0.01) and Time 2’s relative deprivation(β = -0.126, p<0.05). This finding suggests that there is a causal relationship between digital-HRM and sense of work gain, as well as between digital-HRM and relative deprivation, supporting our predictions.

**Table 11:** Results of cross-lagged analysis.

Relationship	Standard Estimate	Standard error	Bias-corrected bootstrap 95% confidence interval	
			Lower	Higher
DHRM(T1)-DHRM(T2)	0.378***	0.052	0.275	0.481
SWG(T1)-SWG(T2)	0.588***	0.053	0.483	0.693
RD(T1)-RD(T2)	0.528***	0.052	0.426	0.629
EIP(T1)-EIP(T2)	0.462***	0.074	0.317	0.607
Cyberloafing(T1)- Cyberloafing(T2)	0.704***	0.036	0.632	0.775
DHRM*PETU(T1)-DHRM*PETU(T2)	0.375***	0.048	0.282	0.468
DHRM(T1)-SWG(T2)	0.191**	0.059	0.075	0.307
SWG(T1)-DHRM(T2)	0.394***	0.064	0.269	0.520
DHRM(T1)-RD(T2)	-0.126*	0.061	-0.246	-0.005
RD(T1)-DHRM(T2)	0.014	0.059	-0.102	0.131
SWG(T1)-EIP(T2)	0.458***	0.096	0.269	0.646
EIP(T1)-SWG(T2)	0.040	0.056	-0.070	0.150
RD(T1)- Cyberloafing(T2)	0.031	0.047	-0.061	0.123
Cyberloafing(T1)-RD(T2)	0.095	0.051	-0.005	0.195
DHRM*PETU(T1)-SWG(T2)	0.151**	0.052	0.049	0.253
SWG(T1)-DHRM*PETU(T2)	-0.163*	0.073	-0.307	-0.019
DHRM*PETU(T1)-RD(T2)	-0.154*	0.060	-0.271	-0.038
RD(T1)-DHRM*PETU(T2)	0.086	0.071	-0.053	0.226

Notes: n = 241

*p<0.05

**p<0.01

***p<0.001.

DHRM = digital-HRM

PETU = perceived ease of technology use

SWG = sense of work gain

RD = relative deprivation

EIP = employee innovative performance

Source: Developed by the authors on the basis of collected data using Smart PLS.

Likewise, after controlling for stability effects (i.e., the impact of sense of work gain at time 1 on sense of work gain at time 2, and the impact of employee innovative performance at time 1 on employee innovative performance at time 2), the impact of sense of work gain at time 1 on employee innovative performance at time 2 was found to be significant(β = 0.458,p<0.001). this indicates that sense of work gain is a cause leading to employee innovative performance, consistent with our predictions. however, after controlling for the stability effects (i.e., the impact of relative deprivation at time 1 on relative deprivation at time 2, and the impact of cyberloafing at time 1 on cyberloafing at time 2), the cross-lagged effect of relative deprivation at time 1 on cyberloafing at time 2 was not significant(β = 0.031,p>0.05), failing to support our prediction.

For the reason behind these findings, we speculate that it may be due to the first data collection occurring at the end of December 2022, during the peak of pandemic control measures. Most employees were in home isolation working online, without supervision from supervisors or colleagues, thus having more freedom and opportunity to engage in cyberloafing. However, the second data collection was conducted at the end of November 2023, when the pandemic control period had ended, and work had returned to normal order. Most employees were working in offices with supervision, significantly reducing the space and freedom for cyberloafing. Even if they felt deprived, they might not dare to engage in cyberloafing. Additionally, this period was marked by economic recovery, with many companies adjusting and getting back on track, requiring employees to perform well to avoid layoffs. Therefore, this could lead to the nonsignificant relationship between relative deprivation and cyberloafing.

Additionally, we have made a new discovery: Time 1’s sense of work gain significantly positively impacts the increase of digital-HRM at Time 2. this suggests that an increase in employee’s sense of work gain can further promote the practice level of digital-HRM in enterprises.

## Discussion

For an extended duration, individuals have harbored apprehensions towards technology, often responding with trepidation whenever novel technological advancements emerge. Nevertheless, historical precedent consistently illustrates the superfluous nature of such fears and anxieties, as said by Scaruffi [132]. It is irrefutable that the advantages ushered in by novel technologies far outweigh the sacrifices they demand. This axiom holds true for contemporary digital human resource management as well.

In the scope of our ongoing investigation, employing a comprehensive and meticulous empirical scrutiny encompassing a cohort of 487 employees hailing from China, we have unearthed a collection of results that prove to be both intriguing and enlightening. These findings exhibit a remarkable alignment with our preliminary anticipations. Our inquiry not only corroborates the efficacy of digital-HRM as an emerging job resource within the digital era, which heeding the entreaty posited by Demerouti [133] for exploration of emerging job resources within the digital era, but also unveils the specific circumstances under which this paradigm of administration is predisposed to yield more favorable consequences. This elucidation holds pivotal significance for organizations.

First, within the context of the interplay between digital-HRM and employee innovative performance, a conspicuous observation is the substantial and affirmative mediating influence exerted by sense of work gain. This discernment imparts noteworthy managerial insights: proficient digital-HRM not only augments the provision of work resources, thereby catering to the requisites of employees, but also augments their sense of work gain, consequently fostering a more favorable ground for the enhancement of their innovative performance. Second, it is also evident that the phenomenon of relative deprivation manifests as a negative mediating factor in the nexus between digital-HRM and cyberloafing conduct. This signifies that digital-HRM not only elicits constructive psychological sentiments and fosters positive occupational conduct, but also serves as a deterrent against adverse sentiments, thereby mitigating counterproductive work comportment. While prevailing discourses regarding digital technology underscore the potential for bias and inequity [134], our investigation proposes that digital-HRM serves as a valuable instrument for mitigating perceptions of injustice, as opposed to functioning as a constraint that fosters such perceptions. It’s worth noting that if employees feel deprived or dissatisfied, the potential negative impact cannot be ignored. This highlights a cautionary consideration for managers: when advocating for the adoption of digital management systems, particular emphasis ought to be placed on addressing the psychological requisites and emotional well-being of employees to preempt the emergence of undesirable behaviors.

In addition to this, we further investigated the impact of perceived ease of technology use on these relationships. Encouragingly, we found that when employees perceived the technology used to be more accessible, digital-HRM had a stronger negative impact on their relative deprivation and cyberloafing, as well as a stronger positive impact on their sense of work gain and employee innovative performance. In fact, this finding is supported by socio-technical systems theory and the technology acceptance model. Socio-technical systems theory emphasizes that an organizational system consists of a technological system and a social system, and the organization can only achieve optimal operational efficiency when these systems are aligned [135]. Applied to our study, this means that organizations can better achieve growth and development through the incorporation of new technologies when employees adapt to and accept these technologies. Moreover, the technology acceptance model also highlights the critical role of perceived ease of use in people’s decision to adopt a technology [136]. Based on this theory, if employees believe that the introduction of new digital technologies simplifies daily workflows and is easy to operate, they are more likely to accept these technologies and actively use them to enhance work efficiency and performance. As a result, in the context of digital-HRM practices, they exhibit more positive psychological emotions and fewer negative emotions. This aligns with the findings of Martínez-Navalón et al. [137], who demonstrated that users’ perceptions of the ease of use of new products significantly positively influence their acceptance.

Furthermore, our study not only focuses on the current effects of digital- HRM but also explores its sustained impact. We conducted follow-up surveys with the same cohort of participants using the cross-lagged panel model approach. Specifically, our findings indicate that in the first phase, digital-HRM significantly enhanced employees’ sense of work gain and substantially reduced their feelings of relative deprivation. These effects were not only significant in the short term but were also confirmed in the second phase of the survey conducted one year later. This demonstrates that digital-HRM has a continuing positive influence on employees’ psychological experiences and helps reduce negative psychological experiences. These findings are crucial for understanding the long-term impacts of digital-HRM, especially regarding employee well-being, retention rates, and organizational performance. They reveal how digital-HRM can improve overall employee well-being by enhancing their psychological state, which directly relates to employees’ retention intentions and individual performance [138, 139]. In the long term, such positive psychological changes can be expected to trigger a positive chain reaction in organizational performance, as improved employee well-being and satisfaction are typically associated with higher productivity [140].

Taking all the above findings together, our study reaffirms the status of digital-HRM as an emerging job resource capable of catalyzing advancement within both organizational and societal contexts, rather than instigating adversity. Concurrently, we underscore the imperative for organizations, amidst the epoch of rapid digital progression, to direct their focus not only towards the technological dimensions them-selves but also towards the perceived and psychological disposition of their workforce. In light of this, we advocate for heightened organizational dedication to the cultivation of employees’ digital proficiency and technological self-efficacy. This concerted effort is poised not solely to augment employee receptivity towards the digital work environment but also to potentially amplify their occupational efficiency and contentment, consequently fostering a more profound contribution to the trajectory of sustainable organizational advancement.

### Theoretical implications

Our study makes three significant theoretical contributions. Firstly, our study responds to the academic call to identify and understand the newly emerging job resources in contemporary work environments [133]. The mainstream perspectives on digitalization and digital technologies often associate them with new forms of Tayloristic management, such as digital control, algorithmic supervision, and algorithm management [12]. In contrast, our research utilizes the job demands-resources theory as a framework, revealing the unique role and potential value of digital-HRM in actual work settings. We emphasize that digital-HRM is an emerging job resource adapted for the digital era, not an oppressive job demand. Specifically, digital-HRM meets employees’ needs for flexibility, skill development, and personalized services by providing a digitalized workplace, digital employee services, and digital HRM processes. This not only enriches the existing literature on the job demands-resources theory but also expands the scope of what constitutes a job resource, thereby aiding in enhancing employee engagement and performance.

Secondly, from a people-centered perspective, we explored the mechanisms by which digital-HRM affects employee-level factors (employee innovation performance and cyberloafing) based on the HRM-WB-OIP theoretical model. We verified that digital-HRM influences employee psychology in a mutually beneficial manner, thereby enhancing performance outcomes. This is the first empirical paper to validate how digital-HRM enhances performance by modulating employees’ psychological states, theoretically expanding the research on digital-HRM in an individual context and contributing to the existing literature on digital-HRM. In recent years, digital-HRM has garnered significant academic attention. Most existing studies discuss digital-HRM from the perspective of “things”, such as the conceptual framework of digital-HRM [53], its implementation paths [33], or the consequences of digital HRM at an organizational level, like organizational performance [37] and productivity of HR functions [36]. However, surprisingly, few studies focus on the impact of digital-HRM on individual-level factors, neglecting attention to the “people”, who are the main subjects of digital-HRM. While some literature has explored the impact of digital-HRM on employee performance [35], it is noteworthy that technology has social attributes [141]. If the implementation of digital-HRM focuses solely on employee performance without considering changes in their psychological states or exploring how to help employees better adapt to digital-HRM, it may not only damage the relationship between the organization and its employees [142] but also hinder the success of digital-HRM, ultimately affecting organizational development negatively [143]. Therefore, it is imperative to deeply understand how digital-HRM influences employee performance by affecting their psychological states. The introduction of the job demands-resources theory offers a new perspective. Relying on this theory, our study posits that digital-HRM is an emerging job resource, not a job demand. It empirically validates that digital-HRM impacts employees’ psychological states and behaviors in a mutually beneficial manner. Specifically, digital-HRM enhances positive psychological experiences (i.e., sense of work gain) and reduces negative psychological experiences (i.e., feelings of relative deprivation), boosting employees’ innovative performance and mitigating cyberloafing behaviors. This deepens our understanding of how digital-HRM serves as a critical factor influencing changes in employee psychology and behavior. This understanding is crucial for organizations, as it guides them on how to more effectively design and implement digital-HRM strategies to promote employee psychological well-being and the long-term health of the organization. Additionally, by introducing the concept of “sense of work gain [65]”—a derivative from a Chinese indigenous psychological framework—our study not only aligns more closely with the Chinese context but also significantly expands the theoretical horizon of digital-HRM, revealing how it enhances employee well-being and drives performance improvements. This also enriches the global discussion on the intersection of technology and employee psychology.

Thirdly, our study contributes to understanding the boundary conditions that affect the effectiveness of digital technology-related job resources, particularly those related to employees’ individual capabilities and beliefs. Our findings indicate that employees’ perceived ease of technology use are a critical factor influencing how they utilize and respond to digital-HRM. Employees with a higher perception of technology ease of use are more likely to actively accept and utilize these resources, as they believe that technology simplifies workflows, enhances work efficiency, and reduces stress caused by technological complexity [144]. Consequently, these employees exhibit a greater sense of work gain and a lesser feeling of relative deprivation when interacting with digital-HRM practices. By identifying this boundary condition, our study enhances the exploration of factors affecting the effectiveness of digital technology-related job resources and offers a new theoretical perspective on the relationship between digital-HRM and employees’ psychological responses. Our results show that when employees have a positive perception of technology ease of use, the relationship between digital-HRM and their sense of work gain and relative deprivation becomes more pronounced, providing crucial insights for organizations in designing and implementing digital-HRM strategies.

### Practical implications

Our research also has significant practical implications for organizations. First, we suggest that organizations consider the benefits of digital transformation and carefully evaluate the implementation of digital-HRM initiatives based on their specific organizational context and readiness. The field of HRM is undergoing rapid changes, and it is increasingly important for HRM not just to serve as a support function, but to actively participate in shaping organizational strategies through digitalization [40]. Organizations are therefore encouraged to develop digital and intelligent HRM systems tailored to their unique needs, which can maximize their potential to enhance employee performance and offer employees greater opportunities for growth. This thoughtful and strategic approach will enable organizations to effectively navigate the evolving competitive landscape and maintain their long-term competitiveness.

Second, from an HR perspective, the increasing adoption of digital technology enables HR professionals to execute various HRM functions more efficiently and swiftly. This encompasses activities such as recruitment processes, training programs, job evaluation, performance measurement, compensation management, and employment relations [39]. To effectively facilitate HRM processes and digitalization, future HR managers will need to acquire digital knowledge, skills, and competencies. This will enable them to proficiently identify and match talent, utilize digital tools like talent portraits and talent dashboards, and provide technical support for talent acquisition, growth, and development within the organization, Such as IBM’s application Watson Recruitment, which recommends the best candidate for any job based on job requirements, Blue Match software, which drives employee career development by algorithmically providing each employee with suggestions for career advancement and new jobs and MYCA, which helps employees identify directions for skill enhancement [3], in addition to Deloitte’s talent management system [145], and AsiaInfo’s Talent Training Programme [146]. Furthermore, to ensure the success of digital transformation initiatives, it is crucial to fully meet the needs of employees [147]. When integrating new digital and artificial intelligence technologies, the human resources department should design and implement a range of targeted technology training programs, such as skill enhancement, online interactive courses, and certification training, to accommodate the learning preferences and career development needs of various employees. Regular, interactive training sessions can help employees demystify these technologies, reducing uncertainty and resistance to new systems. This approach not only helps employees perceive digital tools as enablers rather than threats and builds trust with managers [1] but also stimulates a positive attitude towards technological changes, enabling them to fully realize their potential in new work models. Additionally, organizations can foster a digital-centric corporate culture through cultural activities and innovative training programs, which will aid in developing digital mindsets, innovation awareness, and team collaboration spirit among employees. This encourages them to proactively adapt to changes, actively explore new working methods, and promote ongoing innovation and improvement within the organization.

Last, with regards to individual employees, the digitization of HRM is poised to transform employee-related functions, such as recruitment, training, and assessment, necessitating a profound comprehension of the ensuing changes [40]. It is incumbent upon employees to proactively cultivate relevant digital technology competencies, nurture a sense of self-efficacy in utilizing digital technology, foster a mindset of empowerment rather than being subject to digital control, and be prepared for long-term collaboration with digital technology and AI. Rather than evading or shunning these advancements, employees should embrace this challenge and equip themselves accordingly.

## Conclusions

The present study investigated employees’ responses to the use of digital-HRM in organizations. By drawing upon the job demands-resources theory, we built a dual-pathway theoretical model to examine the mixed effects for employees of digital-HRM. Our hypothesized model was supported by a time-lagged data analysis study. Our findings indicated that digital-HRM promotes employee innovative performance via enhanced sense of work gain, while digital-HRM inhibits cyberloafing via attenuated relative deprivation. Furthermore, both the indirect paths are enhanced by perceived ease of technology use. Our work thus contributes to the literature on digital-HRM by exploring employees’ responses to the practice of digital-HRM and the boundary conditions of such responses.

## Limitations and future research

Firstly, the data used in this paper were collected based on self-reporting, which may affect the objectivity and validity of the data to a certain extent, and future research can adopt multisource data in order to hypothesize results more comprehensive and accurate validation.

Secondly, this study focuses on the mediating role of sense of work gain and relative deprivation without considering other variables. Future research can also refine the mediating mechanisms and boundary conditions of digital human resource management on employees’ innovative performance and cyberloafing from multiple perspectives in order to construct a more systematic and comprehensive theoretical model. Furthermore, in our study, we only focused on employee innovative performance and cyberloafing. While this adds to the existing research, it indeed represents a limitation of our study as it only explores the impact on specific behaviors. Future research could further investigate the effects of digital-HRM on other employee behaviors, for example, examining the impact of digital-HRM practices on employee engagement and burnout based on the job demands-resources theory. Additionally, digital-HRM digitizes the workplace, which raises questions about its impact on colleague relationships and how it shapes employees’ work styles and collaborative tendencies. Future studies could consider these aspects to more comprehensively assess the impact of digital-HRM on employee behavior. Meanwhile, our research shows that perceived ease of technology use moderate the relationship between digital-HRM and employee psychological states. Future research could explore how other individual characteristics or contextual factors influence employees’ responses to digital-HRM practices, such as digital literacy, openness to change, or organizational culture.

Thirdly, the long-term impacts of digital-HRM on employees and organizations merit further exploration. Although current research utilizing cross-lagged panel testing has identified the long-term effects of digital-HRM on employees’ sense of work gain and feelings of relative deprivation, further empirical testing is needed on its long-term effects on employee well-being, retention rates, and organizational performance. Future studies could focus on a detailed exploration and testing of the potential long-term impacts of digital-HRM on employees and organizations. This includes examining the effects on employee well-being, retention rates, and organizational performance, as well as how organizations can strategically implement and optimize digital-HRM to maximize its long-term benefits. Additionally, future research could investigate how to adapt and apply these findings across different types of organizations and cultural contexts.

Finally, conducting qualitative research helps us to gain a more comprehensive understanding of the specific practical effects of digital-HRM and the genuine feelings of employees. Future research could include increasing interviews with HR professionals or general employees to collect information on the application of digital-HRM practices in actual processes, as well as employees’ perceptions and feedback on these practices. This approach would make research outcomes more closely align with the complexity and dynamism of real work environments, complementing and verifying existing quantitative research findings from a practical perspective.

## Supporting information

S1 Data (XLSX)

S1 Appendix (DOCX)

## References

[1] Kambur E, Yildirim T. From traditional to smart human resources management. Int J Manpower. 2023;44(3):422–52.

[2] Strohmeier S. Digital human resource management: A conceptual clarification. German Journal of Human Resource Management. 2020;34(3):345–65.

[3] Li Y, Li L, Hu X. Digital Human Resource Management: Integration Framework and Research Prospects. Science & Technology Progress and Policy. 2021;38:151–60. [IN CHINESE]

[4] Thite M. Electronic/digital HRM: a primer. e-HRM: Routledge; 2018. p. 1–21.

[5] Shah SGS, Nogueras D, van Woerden HC, Kiparoglou V. The effectiveness of digital technology interventions to reduce loneliness in adult people: A protocol for a systematic review and meta-analysis. Medrxiv. 2019:19000414. 10.1136/bmjopen-2019-032455 PMC6773278 31562164

[6] Sheng N, Fang Y, Shao Y, Alterman V, Wang M. The impacts of digital technologies on successful aging in non-work and work domains: An organizing taxonomy. Work, Aging and Retirement. 2022;8(2):198–207.

[7] Wang L, Chen Y, Ramsey TS, Hewings GJ. Will researching digital technology really empower green development? Technol Soc. 2021;66:101638.

[8] Huang S, Long L, Wu D. The perils and opportunities of happiness: the double-edged sword of digital HRM. Tsinghua Business Review. 2022(09):88–99. [IN CHINESE]

[9] Huu PT. Impact of employee digital competence on the relationship between digital autonomy and innovative work behavior: a systematic review. Artif Intell Rev. 2023:1–30. doi: 10.1007/s10462-023-10492-6 37362897 PMC10148002

[10] Rabl T, Petzsche V, Baum M, Franzke S. Can support by digital technologies stimulate intrapreneurial behaviour? The moderating role of management support for innovation and intrapreneurial self-efficacy. Inform Syst J. 2023;33(3):567–97.

[11] Abraham M, Niessen C, Schnabel C, Lorek K, Grimm V, Möslein K, et al. Electronic monitoring at work: The role of attitudes, functions, and perceived control for the acceptance of tracking technologies. Hum Resour Manag J. 2019;29(4):657–75.

[12] Kellogg KC, Valentine MA, Christin A. Algorithms at work: The new contested terrain of control. Acad Manag Ann. 2020;14(1):366–410.

[13] Wang H, Ding H, Kong X. Understanding technostress and employee well-being in digital work: the roles of work exhaustion and workplace knowledge diversity. Int J Manpower. 2023;44(2):334–53.

[14] Wood AJ, Graham M, Lehdonvirta V, Hjorth I. Good gig, bad gig: autonomy and algorithmic control in the global gig economy. Work, Employment and Society. 2019;33(1):56–75. doi: 10.1177/0950017018785616 30886460 PMC6380453

[15] Marsh E, Vallejos EP, Spence A. The digital workplace and its dark side: An integrative review. Comput Hum Behav. 2022;128:107118.

[16] Newman DT, Fast NJ, Harmon DJ. When eliminating bias isn’t fair: Algorithmic reductionism and procedural justice in human resource decisions. Organ Behav Hum Dec. 2020;160:149–67.

[17] Gagné M, Parent-Rocheleau X, Bujold A, Gaudet M, Lirio P. How algorithmic management influences worker motivation: A self-determination theory perspective. Canadian Psychology/Psychologie Canadienne. 2022;63(2):247.

[18] Lang JJ, Yang LF, Cheng C, Cheng XY, Chen FY. Are algorithmically controlled gig workers deeply burned out? An empirical study on employee work engagement. Bmc Psychol. 2023;11(1):354. doi: 10.1186/s40359-023-01402-0 37876010 PMC10598991

[19] Liu N, Wang Y, Lin Y. Employees’ Adaptation to Technology Uncertainty in the Digital Era: An Exploration Through the Lens of Job Demands–Resources Theory. Ieee T Eng Manage. 2023.

[20] Peccei R, Van De Voorde K. Human resource management–well-being–performance research revisited: Past, present, and future. Hum Resour Manag J. 2019;29(4):539–63.

[21] Guest DE. Human resource management and employee well-being: Towards a new analytic framework. Hum Resour Manag J. 2017;27(1):22–38.

[22] Jo H, Aryee S, Hsiung HH, Guest D. Fostering mutual gains: Explaining the influence of high-performance work systems and leadership on psychological health and service performance. Hum Resour Manag J. 2020;30(2):198–225.

[23] Hayat A, Afshari L. Supportive organizational climate: a moderated mediation model of workplace bullying and employee well-being. Pers Rev. 2021;50(7/8):1685–704.

[24] Guerci M, Hauff S, Gilardi S. High performance work practices and their associations with health, happiness and relational well-being: are there any tradeoffs? The International Journal of Human Resource Management. 2022;33(2):329–59.

[25] Okros N, Virga D. Impact of workplace safety on well-being: the mediating role of thriving at work. Pers Rev. 2023;52(7):1861–77.

[26] Pelly D. Worker well-being and quit intentions: is measuring job satisfaction enough? Soc Indic Res. 2023;169(1):397–441.

[27] Zheng H, Vatsa P, Ma W, Zhou X. Working hours and job satisfaction in China: A threshold analysis. China Econ Rev. 2023;77:101902.

[28] Liang M, Xin Z, Yan DX, Jianxiang F. How to improve employee satisfaction and efficiency through different enterprise social media use. J Enterp Inf Manag. 2021;34(3):922–47.

[29] Liu P, Zhang F, Liu Y, Liu S, Huo C. Enabling or burdening?—The double-edged sword impact of digital transformation on employee resilience. Comput Hum Behav. 2024;157:108220.

[30] Fleischer J, Wanckel C. Job satisfaction and the digital transformation of the public sector: The mediating role of job autonomy. Rev Public Pers Adm. 2023:0734371X221148403.

[31] Deng D, Xu G, Qin C. Influence of employees’ perception of digital transformation meaning on work engagement and well-being. Social Behavior and Personality: An International Journal. 2023;51(9):1–13.

[32] Wu J, Gong X, Liu Y. Research on the influence mechanism of employees’ innovation behavior in the context of digital transformation. Front Psychol. 2022;13:1090961. doi: 10.3389/fpsyg.2022.1090961 36605267 PMC9808053

[33] Bansal A, Panchal T, Jabeen F, Mangla SK, Singh G. A study of human resource digital transformation (HRDT): A phenomenon of innovation capability led by digital and individual factors. J Bus Res. 2023;157:113611.

[34] Nicolás-Agustín Á, Jiménez-Jiménez D, Maeso-Fernandez F. The role of human resource practices in the implementation of digital transformation. Int J Manpower. 2022;43(2):395–410.

[35] Theres C, Strohmeier S. Met the expectations? A meta-analysis of the performance consequences of digital HRM. The International Journal of Human Resource Management. 2023:1–36.

[36] Mosca M. Digitalization of HRM: A study of success factors and consequences in the last decade: University of Twente; 2020.

[37] Vardarlier P. Digital transformation of human resource management: Digital applications and strategic tools in HRM. Digital Business Strategies in Blockchain Ecosystems: Transformational Design and Future of Global Business. 2020:239–64.

[38] Démeijer D. Making digital HRM work: A study in changes in perceived consequences of e-HRM in the past decade.: University of Twente; 2017.

[39] Smirnova AM, Zaychenko IM, Bagaeva IV, ^editors. Formation of requirements for human resources in the conditions of digital transformation of business. International Conference on Digital Technologies in Logistics and Infrastructure (ICDTLI 2019); 2019. Pub Place: Atlantis Press; Year Published.

[40] Zhang J, Chen Z. Exploring Human Resource Management Digital Transformation in the Digital Age. J Knowl Econ. 2023:1–17. 10.1007/s13132-023-01214-y PMC9990565 40479358

[41] Bansal A, Panchal T, ^editors. Training transfer during COVID-19 pandemic: A study of technology adoption in the training programs. Academy of management proceedings; 2022. Pub Place: Academy of Management Briarcliff Manor, NY 10510; Year Published.

[42] Chanana N. The impact of COVID-19 pandemic on employees organizational commitment and job satisfaction in reference to gender differences. J Public Aff. 2021;21(4):e2695. doi: 10.1002/pa.2695 34220346 PMC8236929

[43] Curzi Y, Fabbri T, Scapolan AC, Boscolo S. Performance appraisal and innovative behavior in the digital era. Front Psychol. 2019;10:1659. doi: 10.3389/fpsyg.2019.01659 31379682 PMC6652785

[44] Sangaji RC, Setyaning ANA, Marsasi EG, ^editors. A Literature Review on Digital Human Resources Management Towards Digital Skills and Employee Performance. International Conference on Business and Technology; 2022. Pub Place: Springer; Year Published.

[45] Huyghebaert-Zouaghi T, Morin AJ, Fernet C, Austin S, Gillet N. Longitudinal profiles of work-family interface: Their individual and organizational predictors, personal and work outcomes, and implications for onsite and remote workers. J Vocat Behav. 2022;134:103695.

[46] Jaiswal A, Sengupta S, Panda M, Hati L, Prikshat V, Patel P, et al. Teleworking: role of psychological well-being and technostress in the relationship between trust in management and employee performance. Int J Manpower. 2022.

[47] Bieńkowska A, Koszela A, Sałamacha A, Tworek K. COVID-19 oriented HRM strategies influence on job and organizational performance through job-related attitudes. Plos One. 2022;17(4):e266364. doi: 10.1371/journal.pone.0266364 35417468 PMC9007351

[48] Chambel MJ, Castanheira F, Santos A. Teleworking in times of COVID-19: the role of Family-Supportive supervisor behaviors in workers’ work-family management, exhaustion, and work engagement. The International Journal of Human Resource Management. 2023;34(15):2924–59.

[49] Konuk H, Ataman G, Kambur E. The effect of digitalized workplace on employees’ psychological well-being: Digital Taylorism approach. Technol Soc. 2023;74(C).

[50] Ma L, Zhang X. Effects of work interruption on employees’ work performance: moderating role of social media usage. Inform Technol Peopl. 2023.

[51] Ma L, Zhang X, Wang G. The impact of enterprise social media use on employee performance: A grounded theory approach. J Enterp Inf Manag. 2022;35(2):481–503.

[52] Ma L, Zhang X, Ding X. Enterprise social media usage and knowledge hiding: a motivation theory perspective. J Knowl Manag. 2020;24(9):2149–69.

[53] Chen T, He W, Li W. Analysis on Human Resources Management and Digital Human Resources Management System in the Digital Era. Science and Technology Management Research. 2022;42(22):130–6. [IN CHINESE]

[54] Nonaka I, Konno N. The concept of “Ba”: Building a foundation for knowledge creation. Calif Manage Rev. 1998;40(3):40–54.

[55] Corgnet B, Hernán-González R, McCarter MW. The role of the decision-making regime on cooperation in a workgroup social dilemma: An examination of cyberloafing. Games-Basel. 2015;6(4):588–603.

[56] Liberman B, Seidman G, McKenna KY, Buffardi LE. Employee job attitudes and organizational characteristics as predictors of cyberloafing. Comput Hum Behav. 2011;27(6):2192–9.

[57] Gellmers J, Yan N. Digital Leisure Engagement and Positive Outcomes in the Workplace: A Systematic Literature Review. International Journal of Environmental Research and Public Health. 2023;20(2):1014. doi: 10.3390/ijerph20021014 36673769 PMC9859073

[58] Tandon A, Kaur P, Ruparel N, Islam JU, Dhir A. Cyberloafing and cyberslacking in the workplace: systematic literature review of past achievements and future promises. Internet Res. 2022;32(1):55–89.

[59] Blanchard AL, Henle CA. Correlates of different forms of cyberloafing: The role of norms and external locus of control. Comput Hum Behav. 2008;24(3):1067–84.

[60] Lim VK. The IT way of loafing on the job: Cyberloafing, neutralizing and organizational justice. Journal of Organizational Behavior: The International Journal of Industrial, Occupational and Organizational Psychology and Behavior. 2002;23(5):675–94.

[61] Korzynski P, Protsiuk O. What leads to cyberloafing: the empirical study of workload, self-efficacy, time management skills, and mediating effect of job satisfaction. Behav Inform Technol. 2024;43(1):200–11.

[62] Demerouti E, Bakker AB, Nachreiner F, Schaufeli WB. The job demands-resources model of burnout. J Appl Psychol. 2001;86(3):499. 11419809

[63] Bakker AB, Demerouti E. Job demands–resources theory: Taking stock and looking forward. J Occup Health Psych. 2017;22(3):273. doi: 10.1037/ocp0000056 27732008

[64] Schaufeli WB. Applying the job demands-resources model. Organ Dyn. 2017;2(46):120–32.

[65] Zhu P, Liu J. Employees’ Sense of Work Gain: Structure, Scale Development, Antecedents and Consequences. Human Resources Development of China. 2020;37:65–83. [IN CHINESE]

[66] Yang J, Wang G. Migrant Workers’ Sense of Occupational Gain: Theoretical Construction and Empirical Test. Is-Sues in Agricultural Economy. 2019:108–20. [IN CHINESE]

[67] Fabbri T, Mandreoli F, Martoglia R, Scapolan AC, ^editors. Employee attitudes and (digital) collaboration data: A preliminary analysis in the HRM field. 2019 28th International Conference on Computer Communication and Networks (ICCCN); 2019. Pub Place: IEEE; Year Published.

[68] Anandarajan M, Simmers C, Igbaria M. An exploratory investigation of the antecedents and impact of internet usage: An individual perspective. Behav Inform Technol. 2000;19(1):69–85.

[69] Koay KY. Workplace ostracism and cyberloafing: a moderated–mediation model. Internet Res. 2018;28(4):1122–41.

[70] Zhang J, Akhtar MN, Zhang Y, Sun S. Are overqualified employees bad apples? A dual-pathway model of cyberloafing. Internet Res. 2020;30(1):289–313.

[71] Cascio WF, Montealegre R. How technology is changing work and organizations. Annu Rev Organ Psych. 2016;3:349–75.

[72] Bakker AB, Demerouti E, Sanz-Vergel A. Job demands–resources theory: Ten years later. Annu Rev Organ Psych. 2023;10:25–53.

[73] Zheng J. The Measurement and Status Quo of Chinese Citizens’ Perceived Better Life: The Relationship Among Sense of Gain, Sense of Security and Subjective Being-Well. Cass Journal of Political Science. 2020:89–103. [IN CHINESE]

[74] Wang J, Gu Y, Luo Y, Huang Y, Liao L. The mechanism of the influence of coaching leadership behavior on subordinate’s sense of gain at work. Leadership Org Dev J. 2022;43(4):638–52.

[75] Bakker AB, Xanthopoulou D. Creativity and charisma among female leaders: The role of resources and work engagement. The International Journal of Human Resource Management. 2013;24(14):2760–79.

[76] Bakker AB, Petrou P, Op Den Kamp EM, Tims M. Proactive vitality management, work engagement, and creativity: The role of goal orientation. Applied Psychology. 2020;69(2):351–78.

[77] Hui L, Qun W, Nazir S, Mengyu Z, Asadullah MA, Khadim S. Organizational identification perceptions and millennials’ creativity: testing the mediating role of work engagement and the moderating role of work values. Eur J Innov Manag. 2021;24(5):1653–78.

[78] Neuber L, Englitz C, Schulte N, Forthmann B, Holling H. How work engagement relates to performance and absenteeism: a meta-analysis. Eur J Work Organ Psy. 2022;31(2):292–315.

[79] Thite M. Digital human resource development: where are we? Where should we go and how do we go there? Hum Resour Dev Int. 2022;25(1):87–103.

[80] Scott SG, Bruce RA. Determinants of innovative behavior: A path model of individual innovation in the workplace. Acad Manage J. 1994;37(3):580–607.

[81] Sivathanu B, Pillai R. Smart HR 4.0–how industry 4.0 is disrupting HR. Human Resource Management International Digest. 2018;26(4):7–11.

[82] Xie X, Zuo Y, Hu Q. Human Resources Management in the Digital Era: A Human-Technology Interaction Lens. Journal of Management World. 2021;37(01):200–16. [IN CHINESE]

[83] Majumder S, Mondal A. Are chatbots really useful for human resource management? Int J Speech Technol. 2021:1–9.

[84] Zang S, Ye M. Human resource management in the era of big data. Journal of Human Resource and Sustainability Studies. 2015;3(01):41.

[85] Abdeldayem MM, Aldulaimi SH. Trends and opportunities of artificial intelligence in human resource management: Aspirations for public sector in Bahrain. International Journal of Scientific and Technology Research. 2020;9(1):3867–71.

[86] Fay D, Shipton H, West MA, Patterson M. Teamwork and organizational innovation: The moderating role of the HRM context. Creat Innov Manag. 2015;24(2):261–77.

[87] Wright TA, Huang CC. The many benefits of employee well-being in organizational research. J Organ Behav. 2012;33(8):1188–92.

[88] Runciman WG. Relative deprivation and social justice: A study of attitudes to social inequality in twentieth-century England. (No Title). 1966.

[89] Smith HJ, Pettigrew TF, Pippin GM, Bialosiewicz S. Relative deprivation: A theoretical and meta-analytic review. Pers Soc Psychol Rev. 2012;16(3):203–32. doi: 10.1177/1088868311430825 22194251

[90] Feng L, Zhong H. Interrelationships and methods for improving university students’ sense of gain, sense of security, and happiness. Front Psychol. 2021;12:729400. doi: 10.3389/fpsyg.2021.729400 34630241 PMC8497965

[91] Bakker AB, Demerouti E, Euwema MC. Job resources buffer the impact of job demands on burnout. J Occup Health Psych. 2005;10(2):170. doi: 10.1037/1076-8998.10.2.170 15826226

[92] Ong WJ, Johnson MD. Toward a configural theory of job demands and resources. Acad Manage J. 2023;66(1):195–221.

[93] Liang X, Guo G, Shu L, Gong Q, Luo P. Investigating the double-edged sword effect of AI awareness on employee’s service innovative behavior. Tourism Manage. 2022;92:104564.

[94] Stiles BL, Liu X, Kaplan HB. Relative deprivation and deviant adaptations: The mediating effects of negative self-feelings. J Res Crime Delinq. 2000;37(1):64–90.

[95] Zheng X, Jing C, Liu Y, Zhang YY. Why are people ‘Lying Flat’? Personal relative deprivation suppresses self-improvement motivation. Brit J Soc Psychol. 2023;62(2):932–48. doi: 10.1111/bjso.12611 36453146

[96] Qian D, Yun T, Hua W, Yongxin Z, Zongkui Z. The relationship between relative deprivation and online gaming addiction in college students: A moderated mediation model. Acta Psychol Sin. 2018.

[97] Greenberg J. Employee theft as a reaction to underpayment inequity: The hidden cost of pay cuts. occupational crime: Routledge; 2018. p. 99–106.

[98] Ma L, Zhang X, Yu P. Enterprise social media usage and social cyberloafing: an empirical investigation using the JD-R model. Internet Res. 2023.

[99] Rawashdeh AM, Elayan MB, Alhyasat W, Shamout MD. Electronic human resources management perceived usefulness, perceived ease of use and continuance usage intention: the mediating role of user satisfaction in Jordanian hotels sector. Int J Qual Res. 2021;15(2):679.

[100] Wu C, Kuo Y, Wu S. Investigating the antecedents of university students’ behavioral intention to use iPad for learning. International Journal of E-Education, E-Business, E-Management and E-Learning. 2013;3(6):468.

[101] Davis FD. Perceived usefulness, perceived ease of use, and user acceptance of information technology. Mis Quart. 1989:319–40.

[102] Deci EL, Ryan RM. Self-determination theory. Handbook of Theories of Social Psychology. 2012;1(20):416–36.

[103] Deci EL, Connell JP, Ryan RM. Self-determination in a work organization. J Appl Psychol. 1989;74(4):580.

[104] Roca JC, Gagné M. Understanding e-learning continuance intention in the workplace: A self-determination theory perspective. Comput Hum Behav. 2008;24(4):1585–604. http://doi.org/10.1016/j.chb.2007.06.001

[105] Deci EL, Ryan RM, Gagné M, Leone DR, Usunov J, Kornazheva BP. Need satisfaction, motivation, and well-being in the work organizations of a former eastern bloc country: A cross-cultural study of self-determination. Pers Soc Psychol B. 2001;27(8):930–42.

[106] Joo YJ, So H, Kim NH. Examination of relationships among students’ self-determination, technology acceptance, satisfaction, and continuance intention to use K-MOOCs. Comput Educ. 2018;122:260–72.

[107] Engström J, Elg M. A self-determination theory perspective on customer participation in service development. J Serv Mark. 2015;29(6/7):511–21.

[108] Deci EL, Ryan RM. Intrinsic motivation and self-determination in human behavior.: Springer Science & Business Media; 2013.

[109] Ryan RM, Deci EL. Self-determination theory: Basic psychological needs in motivation, development, and wellness.: Guilford publications; 2017.

[110] Xanthopoulou D, Bakker AB, Demerouti E, Schaufeli WB. Reciprocal relationships between job resources, personal resources, and work engagement. J Vocat Behav. 2009;74(3):235–44.

[111] Sergis S, Sampson DG, Pelliccione L. Investigating the impact of Flipped Classroom on students’ learning experiences: A Self-Determination Theory approach. Comput Hum Behav. 2018;78:368–78.

[112] Rezvani A, Khosravi P, Dong L. Motivating users toward continued usage of information systems: Self-determination theory perspective. Comput Hum Behav. 2017;76:263–75.

[113] Podsakoff PM, MacKenzie SB, Lee J, Podsakoff NP. Common method biases in behavioral research: a critical review of the literature and recommended remedies. J Appl Psychol. 2003;88(5):879. doi: 10.1037/0021-9010.88.5.879 14516251

[114] Martin L, Hauret L, Fuhrer C. Digitally transformed home office impacts on job satisfaction, job stress and job productivity. COVID-19 findings. Plos One. 2022;17(3):e265131. doi: 10.1371/journal.pone.0265131 35271671 PMC8912217

[115] Chen X, Al Mamun A, Hussain WMHW, Jingzu G, Yang Q, Shami SSAA. Envisaging the job satisfaction and turnover intention among the young workforce: Evidence from an emerging economy. Plos One. 2023;18(6):e287284. 10.1371/journal.pone.0287284 PMC10275453 37327240

[116] Zhu J, Zhang B, Xie M, Cao Q. Digital leadership and employee creativity: The role of employee job crafting and person-organization fit. Front Psychol. 2022;13:827057. doi: 10.3389/fpsyg.2022.827057 35615170 PMC9125204

[117] Shao Z, Li X, Luo Y, Benitez J. The differential impacts of top management support and transformational supervisory leadership on employees’ digital performance. Eur J Inform Syst. 2022:1–27.

[118] Petzsche V, Rabl T, Franzke S, Baum M. Perceived gain or loss? How digital affordances influence employee corporate entrepreneurship participation likelihood. Eur Manag Rev. 2023;20(2):188–209.

[119] Cho B, Lee D, Kim K. How does relative deprivation influence employee intention to leave a merged company? The role of organizational identification. Hum Resour Manage-Us. 2014;53(3):421–43.

[120] Blau G, Yang Y, Ward-Cook K. Testing a measure of cyberloafing. Journal of Allied Health. 2006;35(1):9–17. 16615292

[121] Janssen O, Van Yperen NW. Employees’ goal orientations, the quality of leader-member exchange, and the outcomes of job performance and job satisfaction. Acad Manage J. 2004;47(3):368–84.

[122] Hayes AF. Introduction to mediation, moderation, and conditional process analysis: A regression-based approach.: Guilford publications; 2013.

[123] Kock N, Lynn G. Lateral collinearity and misleading results in variance-based SEM: An illustration and recommendations. J Assoc Inf Syst. 2012;13(7).

[124] Harman HH. Modern factor analysis.: University of Chicago press; 1960.

[125] Nunnally JC. Assessment of reliability. Psychometric Theory. 1967:206–35.

[126] Fornell C, Larcker DF. Evaluating structural equation models with unobservable variables and measurement error. J Marketing Res. 1981;18(1):39–50.

[127] Hair J Jr, Hair JF Jr, Hult GTM, Ringle CM, Sarstedt M. A primer on partial least squares structural equation modeling (PLS-SEM).: Sage publications; 2021.

[128] Henseler J, Ringle CM, Sarstedt M. A new criterion for assessing discriminant validity in variance-based structural equation modeling. J Acad Market Sci. 2015;43:115–35.

[129] Sheather S. A modern approach to regression with R.: New York, United States: Springer Press; 2009.

[130] Baron RM, Kenny DA. The moderator–mediator variable distinction in social psychological research: Conceptual, strategic, and statistical considerations. J Pers Soc Psychol. 1986;51(6):1173. doi: 10.1037//0022-3514.51.6.1173 3806354

[131] Liu L, Zhang C. Linking environmental management accounting to green organisational behaviour: The mediating role of green human resource management. Plos One. 2022;17(12):e279568. doi: 10.1371/journal.pone.0279568 36576939 PMC9797071

[132] Scaruffi P. human2.0.; 2017.

[133] Demerouti E. Turn digitalization and automation to a job resource. Applied Psychology. 2022;71(4):1205–9.

[134] Velicu A, Barbovschi M, Rotaru I. Socially isolated and digitally excluded. A qualitative exploratory study of the lives of Roma teenage mothers during the COVID-19 lockdown. Technol Soc. 2022;68:101861.

[135] Appelbaum SH. Socio-technical systems theory: an intervention strategy for organizational development. Manage Decis. 1997;35(6):452–63.

[136] Yang H, Yoo Y. It’s all about attitude: revisiting the technology acceptance model. Decis Support Syst. 2004;38(1):19–31.

[137] Martínez-Navalón J, Fernández-Fernández M, Alberto FP. Does privacy and ease of use influence user trust in digital banking applications in Spain and Portugal? Int Entrep Manag J. 2023;19(2):781–803.

[138] Rothausen TJ, Henderson KE, Arnold JK, Malshe A. Should I stay or should I go? Identity and well-being in sensemaking about retention and turnover. J Manage. 2017;43(7):2357–85.

[139] Wright TA, Cropanzano R. Psychological well-being and job satisfaction as predictors of job performance. J Occup Health Psych. 2000;5(1):84. doi: 10.1037//1076-8998.5.1.84 10658888

[140] Van De Voorde K, Paauwe J, Van Veldhoven M. Employee well-being and the HRM–organizational performance relationship: a review of quantitative studies. Int J Manag Rev. 2012;14(4):391–407.

[141] Suchman LA. Human-machine reconfigurations: Plans and situated actions.: Cambridge university press; 2007.

[142] Lee Y. Employees’ negative megaphoning in response to organizational injustice: The mediating role of employee–organization relationship and negative affect. J Bus Ethics. 2022;178(1):89–103.

[143] Koh WL, Yer LK. The impact of the employee-organization relationship on temporary employees’ performance and attitude: testing a Singaporean sample. Int J Hum Resour Man. 2000;11(2):366–87.

[144] Yener S, Arslan A, Kilinç S. The moderating roles of technological self-efficacy and time management in the technostress and employee performance relationship through burnout. Inform Technol Peopl. 2021;34(7):1890–919.

[145] Buckingham M, Goodall A. Reinventing Performance Management. Harvard Bus Rev. 2015;93(4):40–50.

[146] Wang T. AsiaInfo: Practising digital talent management. Harvard Business Review. 2018. [IN CHINESE]

[147] Huang Y. Internal marketing and internal customer: A review, reconceptualization, and extension. Journal of Relationship Marketing. 2020;19(3):165–81.

