# Supplementary material for: The dual path effect mechanism study of digital-HRM on employee innovative performance and cyberloafing
Source: PLoS One. 2024 Aug 15;19(8):e0307195. doi: 10.1371/journal.pone.0307195 (PMC11326615; doi:10.1371/journal.pone.0307195)
Supplement: S1 Appendix — (DOCX) [file pone.0307195.s002.docx]

**Table A1. Measurement Items.**

| *Digital-HRM* |
| --- |
| My organization has established a digital online training platform. |
| My organization uses digital technology to analyze and make decisions on corporate talent data. |
| My organization uses digital technology to analyze and make decisions on employee training data. |
| My organization uses digital technology to analyze and make decisions on assessment-related data. |
| My organization places digital-HRM strategy at the same level of importance as digital business strategy. |
| My organization considers digital-HRM strategy as one of the methods for creating organizational value. |
| *Perceived ease of technology use* |
| Learning to operate the digital technology is easy for me. |
| I find it easy to get the digital technology to do what I want it to do. |
| It is easy for me to become skillful at using the digital technology. |
| I find the digital technology easy to use. |
| *Sense of work gain* |
| My work has been recognized by my superiors. |
| My organization has recognized my achievements at work. |
| The salary I receive is reasonable. |
| Work has allowed me to become proficient in job knowledge and skills. |
| My workload is relatively fair. |
| The work I do is related to my career aspirations. |
| I am confident in my development within the organization. |
| I am hopeful about my future career development. |
| *Relative deprivation* |
| When I compare what I have with my colleagues, I feel like I’m missing something. |
| When I compare what I have from my job to my colleagues, I feel quite prosperous. |
| When I see my colleagues have more, I feel resentful. |
| When I compare what I have with what my colleagues have, I feel very dissatisfied. |
| *Employee innovative performance* |
| I offer new ideas to improve existing working conditions. |
| I take the initiative to research, learn new work methods, skills, or tools. |
| My new ideas receive approval and endorsement from my superiors. |
| I propose some original solutions to problems. |
| I can present innovative ideas using a systematic approach. |
| I often introduce new work methods to my colleagues. |
| *Cyberloafing* |
| Receiving non-work related emails during working hours. |
| Browsing entertainment-related websites during working hours. |
| Downloading non-work-related information during working hours. |
| Browsing general news websites during working hours. |
| Chatting with others via instant messaging during working hours. |
| Browsing websites unrelated to work during working hours. |
